# Mapping an information design game into an all-pay auction

**DOI:** 10.1007/s10058-023-00334-w

**Published:** 2023-07-20

**Authors:** Oleg Muratov

**Affiliations:** University of Bern, Bern, Switzerland

**Keywords:** All-pay auctions, Contests, Information design, Sender–receiver, Project selection, C72, D80

## Abstract

I formally establish the existence of a mapping between a class of information design games with multiple senders and a class of all-pay auctions. I fully characterize this mapping and show how to use it to find equilibria in the information design game. The mapping allows for a straightforward comparative statics analysis of equilibria in the latter class of games. I use it to study the effect of the tie-breaking rule on the distributions of posteriors and the receiver’s payoff.

## Introduction

Games of information design typically involve an uninformed sender who chooses an experiment structure that induces the receiver to behave in a manner that the sender finds desirable. These games have received considerable attention in the theoretical literature in the past decade. Recent papers (Gentzkow and Kamenica 2016; Boleslavsky and Cotton 2018; Au and Kawai 2019, 2020) have focused on modeling multiple senders to be effectively competing for receivers’ attention. In this paper, I focus on the particular setup of Boleslavsky and Cotton (2018). They study two entrepreneurs competing to persuade an investor to choose their project over a competitor’s one. They characterize the equilibrium of this game and compare it to the outcome when the investor has enough resources to fund both projects. They conclude that the investor is better off under a limited amount of funds unless the prior qualities of projects are very high. They also discuss the similarity between their competitive persuasion game, all-pay auctions, and Colonel Blotto games. Using their model, in this paper, we formally establish and characterize a mapping between a class of information design games with multiple senders and a class of all-pay auctions.

Contests and all-pay auctions have been studied extensively. Baye et al. (1996) fully characterize the equilibria in the all-pay auction with multiple bidders. Clark and Riis (1998) study the case of multiple prizes. Siegel (2009) further develops our understanding of such games by considering asymmetries between the players and allowing for a general class of cost functions.

All-pay auctions have also been widely applied to study various competitive environments. Examples include (i) lobbying and campaign spending (Hillman and Riley 1989; Baye et al. 1993; Che and Gale 1998; Sahuguet and Persico 2006), and (ii) patent and R &D races (Moldovanu and Sela 2003; Che and Gale 2003). The current understanding of all-pay auctions allows us to apply these games to study information design problems.

In this paper, after establishing the mapping between the information design game and the all-pay auction, summarized in Fig. 4, we employ the equilibria of the all-pay auction to characterize the equilibrium behavior in the information design game. Applying the equilibria of all-pay auctions instead of studying the original game simplifies the analysis. It is particularly useful when solving the game under a general tie-breaking rule and determining the effect that the tie-breaking rule has on informativeness and players’ payoffs. This analysis also allows for determining the receiver-preferred tie-breaking rule.

The approach used in this article, of first finding the correspondence between the two classes of games and then using the results from contests to study information design, could be applied to other similar games. In particular, the results from all-pay auctions with multiple asymmetric bidders can be applied to study the generalization of Boleslavsky and Cotton (2018) with three and more asymmetric quality projects. Hence, there is potentially a significant scope for applying the results from all-pay auctions and contests literature to current problems in information design.

## Setup

Consider a model of two entrepreneurs (senders) competing for the funds of a single investor (receiver). The investor has enough money to invest in one indivisible project only. Each entrepreneur has one project idea, good or bad. The qualities of the two projects are independent. Let the ex-ante probability that entrepreneur *i* has a good project be $\alpha _{i,0}\in (0,1)$.

A project requires an investment of $r\in (0,1)$. The good project brings a gross return of 1 to the investor, and the bad project brings 0. If the investor chooses to invest in the project of entrepreneur *i*, that entrepreneur gets a fixed payoff of $w>0$; otherwise, he gets 0. The investor may choose not to invest in any project.

The true qualities of the projects are unknown to any player. The information is symmetric throughout the game. Before the investor chooses the project to invest in (if any), the entrepreneurs simultaneously conduct informative experiments about the qualities of their respective projects. The results of these experiments are public.

It is without loss of generality to formalize entrepreneur *i*’s choice of the experiment as a choice of the distribution of posterior beliefs about the quality of *i*’s project, $G_i({\hat{\alpha }}_i)\in \Delta (\Delta (\{\text {good},\text {bad}\}))$ such that $\int _{0}^{1}{\hat{\alpha }}_i dG_i({\hat{\alpha }}_i)=\alpha _{0,i}$, as in the setting of Kamenica and Gentzkow (2011).

It is also important to discuss the tie-breaking rule. If ${\hat{\alpha }}_1={\hat{\alpha }}_2\geqslant r$, we let the investor break the tie in favor of the entrepreneur *i* with probability $\rho _i\in [0,1]$, $\rho _1+\rho _2=1$. It is suboptimal for the investor to award any contract if posteriors are strictly below *r* and to choose $\rho _1+\rho _2<1$. The tie-breaking rule is a part of the equilibrium since it is the investor’s decision, whom to favor in case of a tie, and the entrepreneurs have to form beliefs about the tie-breaking probabilities. An equilibrium of this game with $\rho _1=1/2$ has been characterized and analyzed in Boleslavsky and Cotton (2018). Our approach will prove essential for generalizing the tie-breaking rule and analyzing its effect on informativeness and the investor’s payoff.

## Analysis

Our focus is to formally establish and characterize a mapping between the equilibria of the information design game, as studied in Boleslavsky and Cotton (2018), and the equilibria of all-pay auctions with a reserve price and a bid cap. This inter-game mapping allows for an alternative way to solve for equilibria of the information design game: our knowledge of outcomes in all-pay auctions transforms into the characterization of the results in the information design game. Moreover, this approach simplifies the comparative statics: changes in the exogenous variables of the information design game affect the auxiliary valuations variables of the all-pay auction game $(V_1, V_2)$, for which we know the effect on the equilibrium. After we characterize the mapping, we apply it to study the comparative statics with respect to the tie-breaking rule. Besides serving as an application of our approach, tie-breaking rule analysis is important in and of itself because we show that the receiver’s payoff is generally higher under non-symmetric tie-breaking.

### Characterizing the mapping

Applying a technique similar to the one used in the appendix of Sahuguet and Persico (2006), consider entrepreneur *i*’s decision when choosing the distribution of posteriors, $G_i$, and fix *i*’s opponent’s distribution of posteriors, $G_k$. Entrepreneur *i*’s optimized payoff at this stage is

ID $$\begin{aligned} W&=\max _{G_i}\int _{0}^{1}w\times ({\mathbb {P}}\{{{\hat{\alpha }}}_k<x\}+\rho _i{\mathbb {P}}\{{{\hat{\alpha }}}_k=x\}){\mathbb {I}}_{\{x\geqslant r\}}d G_i(x) \\&\text {s.t.}\\&\int _{0}^{1}xdG_i(x)=\alpha _{i,0}, \text { and } G_i \text { is a CDF}. \end{aligned}$$

Writing down the Lagrangian that corresponds to this optimization problem, we have 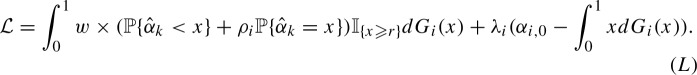 After a series of transformations, we can also write the Lagrangian down as 
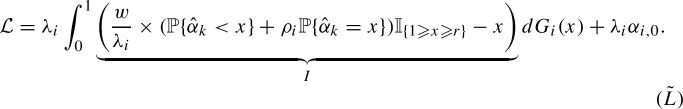
 Consider the integrand *I* of the above expression and, in turn, rewrite it as an integral with respect to the opponent’s CDF as a measure:

$$\begin{aligned} I&=\frac{w}{\lambda _i}\times ({\mathbb {P}}\{{{\hat{\alpha }}}_k<x\}+\rho _i{\mathbb {P}}\{{{\hat{\alpha }}}_k=x\}){\mathbb {I}}_{\{1\geqslant x\geqslant r\}}-x\\&=\int _{0}^{1}\underbrace{\left( \frac{w}{\lambda _i}({\mathbb {I}}_{\{t<x\}}+\rho _i{\mathbb {I}}_{\{t=x\}})-x\right) }_{v_i}dG_k(t). \end{aligned}$$

Without using the indicator functions, write down the latter integrand as

$$\begin{aligned} v_i(x,t)= {\left\{ \begin{array}{ll} \frac{w}{\lambda _i}-x, &{}\quad \text { if }x>t \text { and }x\in [r,1]\\ \rho _i\frac{w}{\lambda _i}-x, &{}\quad \text { if }x=t \text { and }x\in [r,1]\\ -x, &{}\quad \text { if }x<t \text { or }x\notin [r,1]. \end{array}\right. } \end{aligned}$$

Recall that player *i* controls the distribution of *x* and player *k*—that of *t*. In the above expression, we can re-interpret *x* as player *i*’s choice of a bid, $b_i$, and *t* as his opponent’s choice of a bid, $b_k$. Note also that having a higher bid is necessary for winning the prize of value $\frac{w}{\lambda _i}$, that the bid always has to be paid, regardless of winning or losing, and that one can only ever win by bidding above *r* but below 1. We can conclude that this expression coincides with the payoff of a contestant in the all-pay auction with a reserve price *r*, a bid cap of 1, a tie-breaking rule $(\rho _i,\rho _k)$, and a valuation $\frac{w}{\lambda _i}$.

Every equilibrium of the Information Design game includes the pair of CDFs of posterior beliefs, $G_1^*$, $G_2^*$, such that one CDF, $G_i^*$, is the maximizer in the Information Design problem (ID) stated above, taking the other CDF, $G_k^*$, and also the tie-breaking rule, as given. To every pair $(G_1^*,G_2^*)$ corresponds a pair of Lagrange Multipliers, $(\lambda _1^*,\lambda _2^*)$. Besides, every such pair constitutes equilibrium CDFs of bids in the all-pay auction with a reserve price *r*, a bid cap of 1, and a pair of valuations $\left( \frac{w}{\lambda ^*_1},\frac{w}{\lambda ^*_2}\right)$: for both games, $G_i^*$ maximizes the expected payoff given the opponent’s $G_k^*$.

Note that in the corresponding all-pay auction, the equilibrium pair of expected bids equals $(\alpha _{1,0},\alpha _{2,0})$. Let us characterize equilibria in the all-pay auction for all pairs of valuations $(V_1,V_2)$ that result in expected bids falling within the unit square, $(\alpha _{1,0},\alpha _{2,0})\in (0,1)^2$. Doing so allows us to characterize the equilibria in the original information design game. Setting the pair of equilibrium expected bids in the all-pay auction game equal to the pair of prior probabilities in the original information design game would then pinpoint the exact mapping.

### Equilibrium bidding and expenditures

**Fig. 1:**
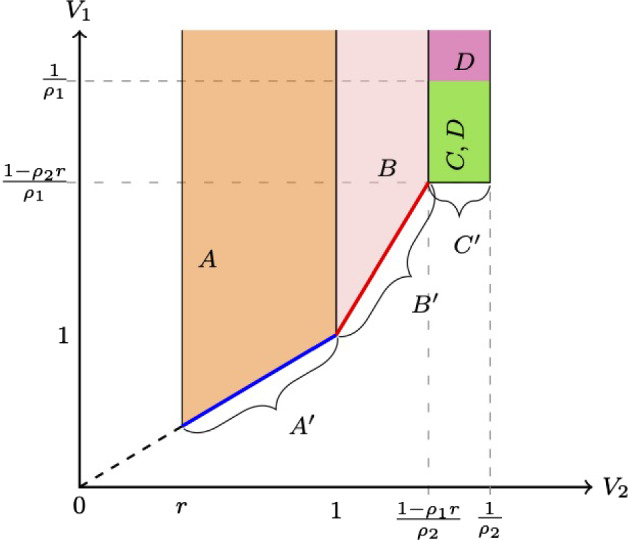
Equilibria across parameter zones, $r=\frac{2}{5}$, $\rho _1=\frac{3}{8}$

Denote player *i*’s valuation from winning the item as $V_i$. In a working paper, Muratov (2022), I find and characterize the equilibria of the all-pay auction with the reserve price and the bid cap.^1^ Figure 1 shows the parameter regions with different equilibrium regimes (note that there is a non-empty subregion with multiple equilibria, indicated by labels *C* and *D*; the multiplicity of equilibria will be further addressed below).

**Fig. 2:**
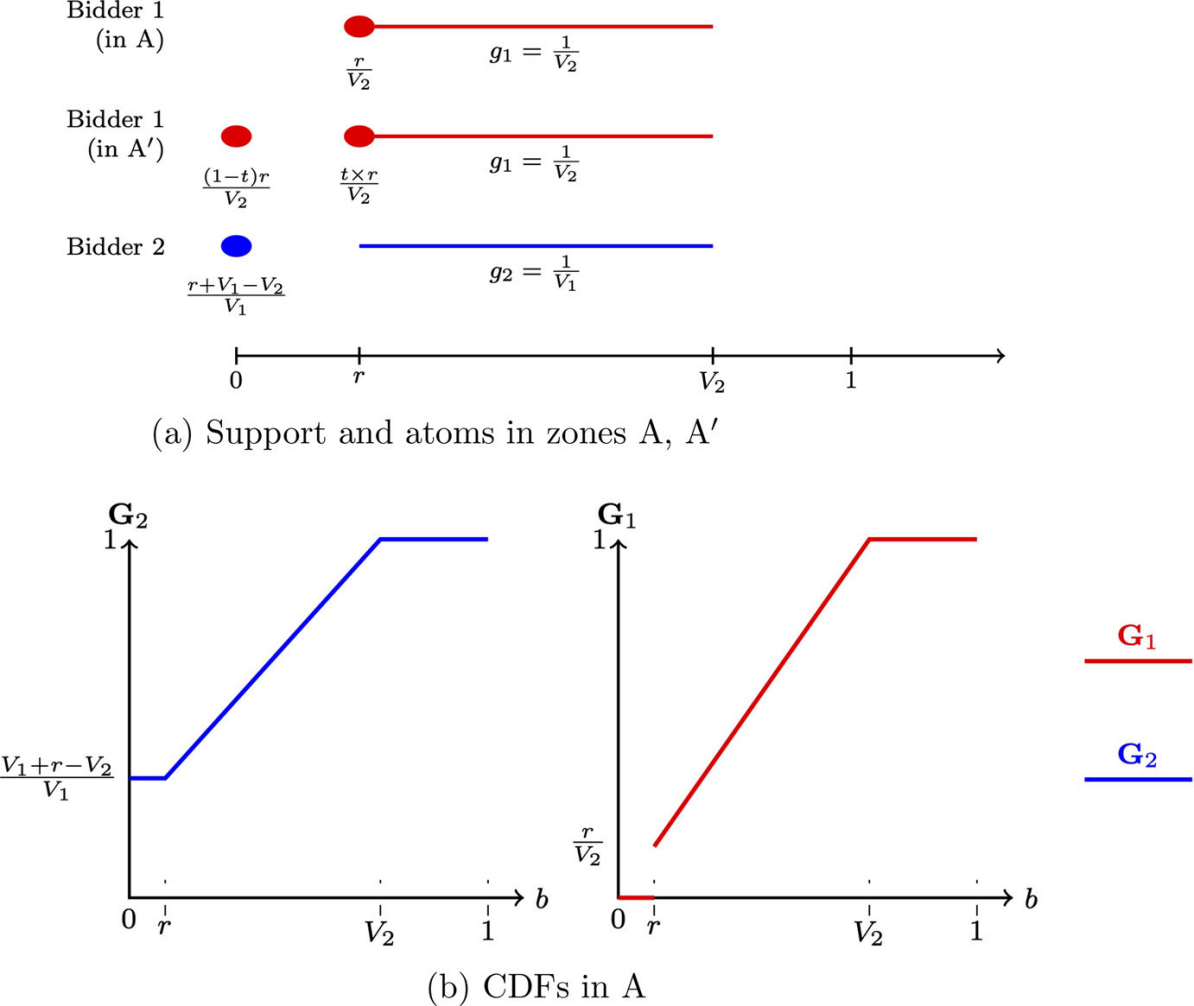
Equilibrium in zones A, A^′^

**Fig. 3:**
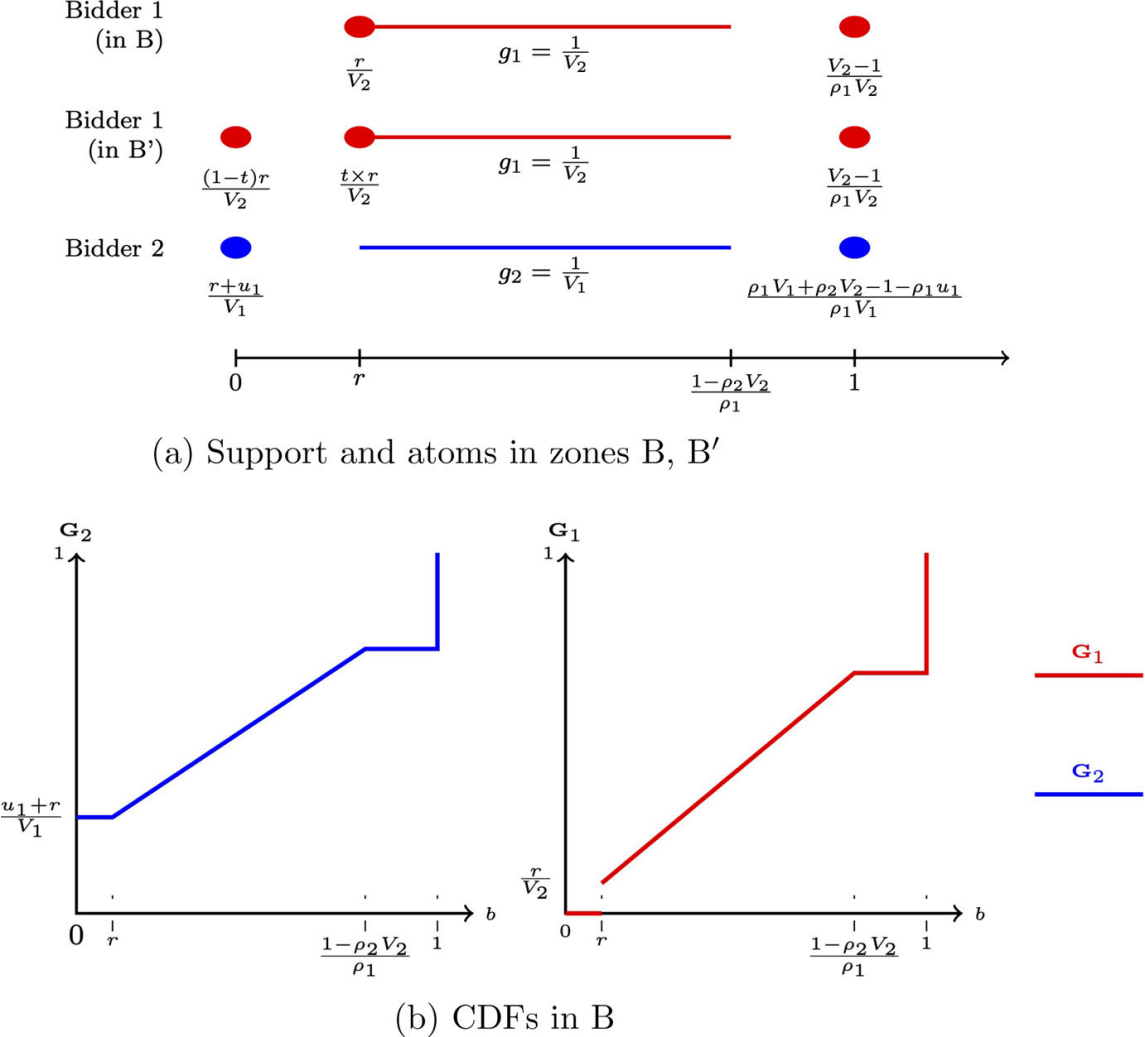
Equilibrium in zones B, B^′^

Below is a brief description of the equilibrium behavior in each

(A, A^′^): For the region $V_1\geqslant V_2\in [r,1)$, Fig. 2a shows the support of equilibrium bidding strategies and the atoms. The dots indicate the atoms and the solid lines indicate the support of continuous bidding.
  Note that bidder 1 has multiple equilibrium strategies in zone A^′^, as $t\in [0,1]$. *t* is a free parameter, with $\frac{t\times r}{V_2}$ standing for the size of the atom that player 1 has at bidding *r*; Fig. 2b plots a typical pair of CDFs for this case.
(*B*, $$B^{\prime }$$)
Zones *B* and $B^{\prime }$ are defined as
  $$\begin{aligned} B(V_1,V_2;\rho _1,\rho _2)&=\left\{ \rho _1^2V_1-\rho _1> \rho _2^2V_2-\rho _2,V_2\in \left[ 1,\frac{1-\rho _1r}{1-\rho _1}\right) \right\} ,\\ B^{\prime }(V_1,V_2;\rho _1,\rho _2&)=\left\{ \rho _1^2V_1-\rho _1=\rho _2^2V_2-\rho _2,V_2\in \left[ 1,\frac{1-\rho _1r}{1-\rho _1}\right) \right\} . \end{aligned}$$
  Figure 3a shows the equilibrium bidding in those regions.^2^ Figure 3b shows a pair of CDFs for a typical value of $(V_1,V_2)$ in the region *B*.
(C): If $(V_1,V_2)\in \left[ \frac{1-\rho _2r}{\rho _1},\frac{1}{\rho _1}\right) \times \left[ \frac{1-\rho _1r}{\rho _2},\frac{1}{\rho _2}\right)$ in an equilibrium player 1 bids 0 and 1 with probabilities $\frac{1-\rho _2V_2}{\rho _1V_2}$ and $\frac{V_2-1}{\rho _1V_2}$, respectively; player 2 bids 0 and 1 with probabilities $\frac{1-\rho _1V_1}{\rho _2V_1}$ and $\frac{V_1-1}{\rho _2V_1}$, respectively.
(C’): If $V_1=\frac{1-\rho _2r}{\rho _1}$, $V_2\in \left[ \frac{1-\rho _1r}{\rho _2},\frac{1}{\rho _2}\right)$, in an equilibrium player 1 bids 0, *r*, and 1, with probabilities $\left( t\frac{1-\rho _2V_2}{\rho _1V_2},(1-t)\frac{1-\rho _2V_2}{\rho _1V_2},\frac{V_2-1}{\rho _1V_2}\right)$, respectively; player 2 bids 0 and 1 with probabilities $\frac{\rho _1r}{1-\rho _2r}$ and $\frac{1-r}{1-\rho _2r}$, respectively.^3^
(D): If $(V_1,V_2)\in \left[ \frac{1-\rho _2r}{\rho _1},+\infty \right) \times \left[ \frac{1-\rho _1r}{\rho _2},\frac{1}{\rho _2}\right)$ in an equilibrium player 1 bids *r* and 1 with probabilities $\frac{1-\rho _2V_2}{\rho _1V_2}$ and $\frac{V_2-1}{\rho _1V_2}$, respectively; player 2 bids 0 and 1 with probabilities $\frac{\rho _1V_1-(1-r)}{\rho _1V_1}$ and $\frac{1-r}{\rho _1V_1}$, respectively.

The remaining cases are either symmetric to the ones described or result in the expenditures being on the boundary of the unit square ($(\alpha _{1,0},\alpha _{2,0})\in [0,1]^2\backslash (0,1)^2$).

**Fig. 4:**
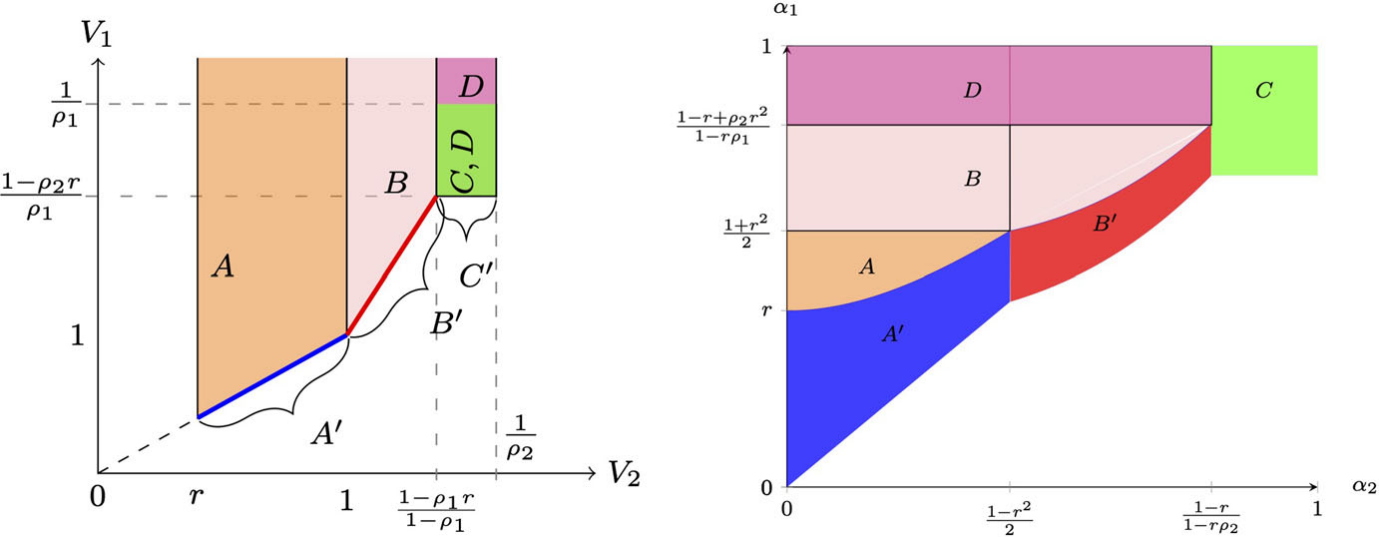
Mapping of equilibria, $r=\frac{2}{5}$, $\rho _1=\frac{3}{8}$

Recall that the prior expected qualities of projects in the information design game correspond to the expected spending of the players in the all-pay auction. Figure 4 shows the correspondence between geometric regions of different types of equilibria in the space of prize valuations $(V_1,V_2)$ and in the space of priors/expenses $(\alpha _{1,0},\alpha _{2,0})$.

Several observations are worth pointing out. First, notice that zones $A^{\prime }$ and $B^{\prime }$ are line segments in the $(V_1,V_2)$-space; thus, corresponding equilibria occur in knife-edge cases. However, in the $(\alpha _{1,0},\alpha _{2,0})$-space, the same zones have non-zero measures. This phenomenon occurs precisely because of the multiplicity of equilibria in $A^{\prime }$ and $B^{\prime }$ of the all-pay auction game. Namely, various sizes of player 1’s atom at *r* lead to multiple levels of $\alpha _{1,0}$ possible for a fixed value of $\alpha _{2,0}$.

Second, note that regions *C* and *D* have a non-empty intersection in the $(V_1,V_2)$-space, while in the $(\alpha _{1,0},\alpha _{2,0})$-space, they only have a knife-edge intersection along $\alpha _{2,0}=\frac{1-r}{1-r\rho _2}$. This happens because *C* and *D* in the all-pay auction lead to different equilibrium bidding and, as a result, different ranges of expenditures, which correspond to non-overlapping (except for the line segment) *C* and *D* in the information design game.

The expressions for the geometric regions of different equilibrium regimes in the $(\alpha _{1,0},\alpha _{2,0})$–space are:

3.1 $$\begin{aligned} A(\alpha _{1,0},\alpha _{2,0})= & {} \left\{ \alpha _{1,0}\in \left[ r,\frac{1}{2}(1+r^2)\right) ,\alpha _{2,0}\in \left[ 0,\sqrt{\alpha _{1,0}^2-r^2}\right) \right\} , \end{aligned}$$

3.2 $$\begin{aligned} A^{\prime }(\alpha _{1,0},\alpha _{2,0})= & {} \left\{ \alpha _{1,0}\in \left[ \alpha _{2,0},\sqrt{\alpha _{2,0}^2+r^2}\right] ,\alpha _{2,0}\in \left[ 0,\frac{1}{2}\left( 1-r^2\right) \right] \right\} , \nonumber \\ \end{aligned}$$

3.3 $$\begin{aligned} B\left( \alpha _{1,0},\alpha _{2,0};\rho _1,\rho _2\right)= & {} \left\{ \frac{1}{2}\right. (1+r^2)\leqslant \alpha _{1,0}<\frac{1-r+\rho _2r^2}{1-\rho _1r}, \nonumber \\{} & {} \quad 0\leqslant \alpha _{2,0}<\left. \frac{(1-2\rho _1+\alpha _{1,0}\rho _1^2)\psi +\psi ^2}{\rho _2^2(\alpha _{1,0}\rho _1^2+\psi )}\right\} , \end{aligned}$$

where $$\psi =\rho _1\sqrt{2\rho _1+\alpha _{1,0}(2-\rho _1(4-\alpha _{1,0}\rho _1))-1-r^2\rho _2^2}$$;

3.4 $$\begin{aligned}{} & {} B^{\prime }\left( \alpha _{1,0},\alpha _{2,0};\rho _1,\rho _2\right) \nonumber \\{} & {} \quad =\left\{ \underline{\alpha _1}\leqslant \alpha _{1,0}\leqslant \underline{\alpha _1}+\frac{r^2\rho _2^2}{\alpha _{2,0}\rho _2^2+\rho _2\phi },\frac{1-r^2}{2}<\alpha _{2,0}\leqslant \frac{1-r}{1-r\rho _2}\right\} , \end{aligned}$$

where

3.5 $$\begin{aligned} {\underline{\alpha }}_1(\alpha _{2,0},\rho _1,\rho _2)= & {} \frac{(\rho _1-\rho _2)\left( (\rho _2-\rho _1)-\rho _2\phi +\rho _1^2r^2+\alpha _{2,0}^2\rho _2^2\right) }{\rho _1^2(1+2\alpha _{2,0}(\rho _1-\rho _2)-\rho _1(2-\rho _1r^2))}\nonumber \\{} & {} \quad +\frac{\alpha _{2,0}(2-\phi -\rho _1(8\rho _2-\rho _1\rho _2^2r^2-(1+2\rho _2)\phi ))}{\rho _1^2(1+2\alpha _{2,0}(\rho _1-\rho _2)-\rho _1(2-\rho _1r^2))},\nonumber \\ \phi= & {} \sqrt{(\rho _2-\rho _1)(1-\alpha _{2,0})^2+\rho _1^2(r^2+\alpha _{2,0}^2)}; \end{aligned}$$

and

3.6 $$\begin{aligned} C(\alpha _1,\alpha _2;\rho _1,\rho _2)= & {} \left\{ \frac{1-r}{1-\rho _1r}\leqslant \alpha _{1,0}<1,\frac{1-r}{1-\rho _2r}\leqslant \alpha _{2,0}<1\right\} , \end{aligned}$$

3.7 $$\begin{aligned} C^{\prime }(\alpha _1,\alpha _2;\rho _1,\rho _2)= & {} \left\{ \frac{1-r}{1-\rho _1r}\leqslant \alpha _{1,0}<1,\alpha _{2,0}=\frac{1-r}{1-\rho _2r}\right\} , \end{aligned}$$

3.8 $$\begin{aligned} D\left( \alpha _{1,0},\alpha _{2,0};\rho _1,\rho _2\right)= & {} \left\{ \frac{1-r+\rho _2r^2}{1-r\rho _1}\leqslant \alpha _{1,0}<1,0\leqslant \alpha _{2,0}\leqslant \frac{1-r}{1-\rho _2r}\right\} .\nonumber \\ \end{aligned}$$

In the expressions above, $\psi$ being properly defined is ensured by $\alpha _{1,0}\geqslant (1/2)(1+r^2)$ and $\phi$ – by $\alpha _{2,0}\geqslant \frac{1-r^2}{2}$.

It is also useful to define how the priors $(\alpha _{1,0},\alpha _{2,0})$ map into the valuations in zones with continuous bidding, i.e. *A*, $A^{\prime }$, *B*, and $B^{\prime }$:

3.9 $$\begin{aligned} V_2^{A}= & {} \alpha _{1,0}+\sqrt{\alpha _{1,0}^2-r^2},V_1^A=\frac{\alpha _{1,0}V_2^A-r^2}{\alpha _{2,0}} \end{aligned}$$

3.10 $$\begin{aligned} V_2^{A^{\prime }}= & {} \alpha _{2,0}+\sqrt{\alpha _{2,0}^2+r^2},V_1^{A^{\prime }}=V_2^{A^{\prime }} \end{aligned}$$

3.11 $$\begin{aligned} V_2^B= & {} \frac{1-\rho _1(2-\alpha _{1}\rho _1)+\psi }{\rho _2^2}, \end{aligned}$$

3.12 $$\begin{aligned} V_1^B= & {} \frac{1-2\rho _1+\alpha _{1,0}\rho _1^2}{\rho _1^2\alpha _{2,0}}V_2^B+\frac{\rho _1(2-\rho _1r^2)-1}{\rho _1^2\alpha _{2,0}},\nonumber \\ V_2^{B^{\prime }}= & {} \frac{\alpha _{2,0}\rho _2^2+\sqrt{\rho _2^2(\rho _2-\rho _1+r^2\rho _1^2-2\alpha _{2,0}(\rho _2-\rho _1))+(\alpha _{2,0}\rho _2^2)^2}}{\rho _2^2}, \nonumber \\ V_1^{B^{\prime }}= & {} \frac{\rho _2^2V_2^{B^{\prime }}+(\rho _1-\rho _2)}{\rho _1^2}. \end{aligned}$$

We can now state the mapping result:

#### Proposition 1

There exists a unique mapping between equilibria in the information design game and the all-pay auction. For every pair of priors in zones *A*–*D* of $(\alpha _{1,0},\alpha _{2,0})$-space, this mapping defines a pair of valuations in a corresponding zone of $(V_1,V_2)$-space. For each zone, the equilibrium distributions of posteriors in the information design game are the same as the equilibrium distributions in the all-pay auction. The mapping is defined by 3.1–3.8 and 3.9–3.12.

We derive the formulae for valuations and the correspondence between the geometric regions of priors and valuations in “Appendix A”.

The algorithm to find the equilibrium in the information design game using the mapping is the following. Given an exogenous pair of priors, $(\alpha _{1,0},\alpha _{2,0})$, and fixing the equilibrium strategy of the investor, summarized by the tie-breaking rule, $(\rho _1,\rho _2)$, we can determine in which region, *A* to *D*, the pair of priors fall. Then, depending on the region, we know what type of equilibrium behavior the bidders follow. The formulae provide the pairs of valuations $(V_1,V_2)$ for each pair of priors. Having the valuations, we can write down the expressions for the equilibrium CDFs.

Using this mapping, we can also perform the comparative statics exercise. Having the auxiliary valuations variables is especially useful for that purpose. From the point of view of the information design game, those variables are endogenous. However, in the all-pay auction game, they are exogenous, and the way the distributions react to changes in valuations is well understood.

*Investor’s payoff* The first–best investor’s payoff occurs under the perfectly informative experiment. In equilibrium, it occurs in zone *C*. The support of posteriors there is $\{0,1\}^2$. In other regions, there are some informational losses.

It is important to notice that in regions $A^{\prime }$ and $B^{\prime }$, if $\alpha _{1,0}$ increases, but the pair of priors stay within the same region, it only results in entrepreneur 1 putting more mass on the atom at *r*. Hence, such an increase in $\alpha _{1,0}$ does not lead to a higher investor’s payoff. However, if both priors increase along the 45-degree line in $A^{\prime }$ or the ${\underline{\alpha }}_{1}(\alpha _{2,0},\rho _1,\rho _2)$-line in $B^{\prime }$, it leads to changes in the supports of posteriors and increases the investor’s payoff. Moreover, along the 45-degree line in $A^{\prime }$ and along the ${\underline{\alpha }}_{1}$-line in $B^{\prime }$, entrepreneur 1 does not have an atom at *r* but has an atom at 0. There is also some continuously distributed mass above *r* but below 1. In zone $B^{\prime }$, as priors approach zone *C* along the ${\underline{\alpha }}_1$-line, the continuous parts of distributions shrink, and the supports become increasingly closer to $\{0,1\}^2$, unlike in zones *B* and *D*, where entrepreneur 1 always has an atom at *r*.

## Tie-breaking rule analysis

In this section, we analyze the effect of varying the tie-breaking rule $(\rho _1,\rho _2)$ in the information design game. Here, we treat the valuations in the all-pay auction preimage game $(V_1,V_2)$ as endogenous and the prior beliefs in the information design game $(\alpha _{1,0},\alpha _{2,0})$ as exogenous.

In zones *A* and $A^{\prime }$, the tie-breaking rule plays no role. In zones *B*, $B^{\prime }$, and *D*, the tie-breaking rule has two effects: it shifts the borders of those regions, and it changes the equilibrium behavior of the entrepreneurs. The tie-breaking rule also affects the borders of *C*.

In the series of lemmata below, we first characterize how the entrepreneurs’ behavior changes with the tie-breaking rule within the zones, and then how the borders of the zones change.

Let  and ${\widehat{\rho }}=(\widehat{\rho _1},\widehat{\rho _2})$ such that . Denote the distributions of posterior beliefs in equilibrium under  by , and under ${\widehat{\rho }}$—by $({\widehat{G}}_{1},{\widehat{G}}_2)$.

### Lemma 1

Choose two tie-breaking rules , ${\widehat{\rho }}$, and priors $(\alpha _{1,0},\alpha _{2,0})$ so that $(\alpha _{1,0},\alpha _{2,0})\in B$ under both tie-breaking rules and . Then, both  and  in the sense of second-order stochastic dominance (hereafter, *SOSD*). The investor prefers the equilibrium under  to that under ${\widehat{\rho }}$.

The proof of the lemma above is in “Appendix B.1”, where we apply the implicit function theorem to the system

$$\begin{aligned} \alpha _{1,0}=\frac{(\rho _2-\rho _1)(V_2-1)^2+\rho _1^2(r^2+V_2^2)}{2V_2\rho _1^2},\; \alpha _{2,0}=\frac{\rho _2^2V_2^2+\rho _1(2-\rho _1r^2)-1}{2V_1\rho _1^2} \end{aligned}$$

to study the effect of change of $\rho _1$ on $V_1$ and $V_2$.^4^ We show that $V_1$ is decreasing in $\rho _1$, and $V_2$ is increasing. After that, knowing how the CDFs depend on $V_1$ and $V_2$, we can conclude the stochastic ordering stated in the lemma.

Recall also that experiment A is Blackwell more informative than experiment B if the distribution of posteriors under B dominates that under A in the sense of *SOSD*. Since both distributions of posteriors are dominated for the lower value of $\rho _1$, they are more informative, and the receiver prefers more informative outcomes.

**Fig. 5:**
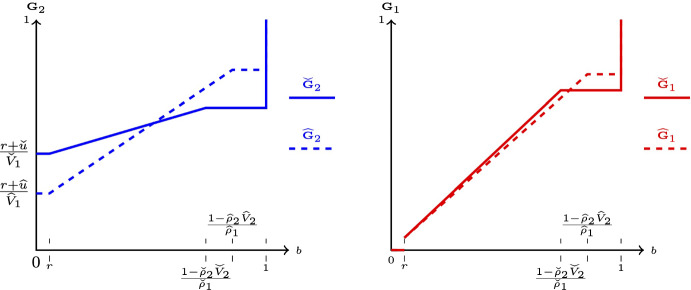
Change of distributions in response to $\rho _1$ increase in *B*

We plot the change of distributions with respect to an increase of $\rho _1$ in zone *B* in Fig. 5.

We continue with the comparative statics with respect to $\rho$ in zone $B^{\prime }$.

### Lemma 2

Choose two tie-breaking rules , ${\widehat{\rho }}$ and priors $(\alpha _{1,0},\alpha _{2,0})$ so that $(\alpha _{1,0},\alpha _{2,0})\in B^{\prime }$ under both tie-breaking rules and . Then, both  and  *SOSD*. The investor prefers the equilibrium under ${\widehat{\rho }}$ to that under .

The proof in “Appendix B.2” again relies on applying the implicit function theorem to the system that defines expenditures in zone $B^{\prime }$ joint with the equation $\rho _1^2V_1-\rho _1=\rho _2^2V_2-\rho _2$.

**Fig. 6:**
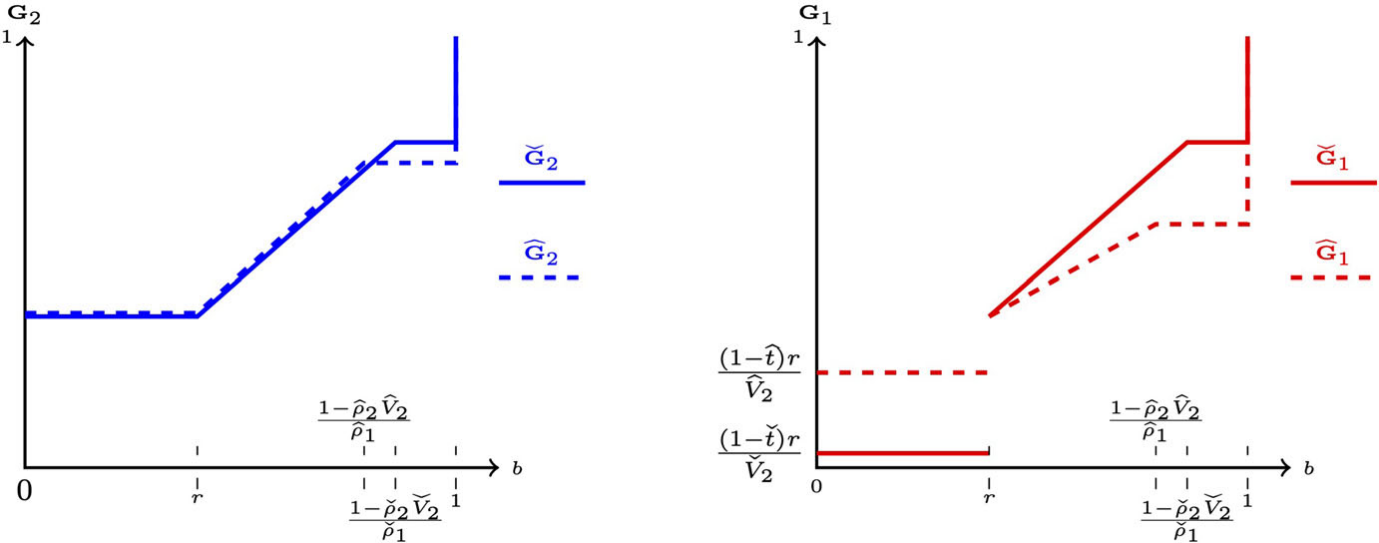
Change of distributions in response to $\rho _1$ increase in $B^{\prime }$

Note that the increase of $\rho _1$ has opposite effects on the investor’s payoff in zones *B* and $B^{\prime }$: the payoff increases with $\rho _1$ in $B^{\prime }$ but decreases in *B* (Fig. 6).

Besides the change of equilibrium behavior of entrepreneurs within the regions, varying the tie-breaking rule also shifts the borders of the regions. The directions of those shifts are stated below:

### Lemma 3

With the increase of $\rho _1$:

(i) The upper bound of *B* on $\alpha _{1,0}$ increases, and the upper bound on $\alpha _{2,0}$ decreases;
(ii) The upper bound of $B^{\prime }$ on $\alpha _{2,0}$ decreases, and both bounds on $\alpha _{1,0}$ increase;
(iii) The upper bound of *D* on $\alpha _{2,0}$ decreases, and the lower bound on $\alpha _{1,0}$ increases.

**Fig. 7:**
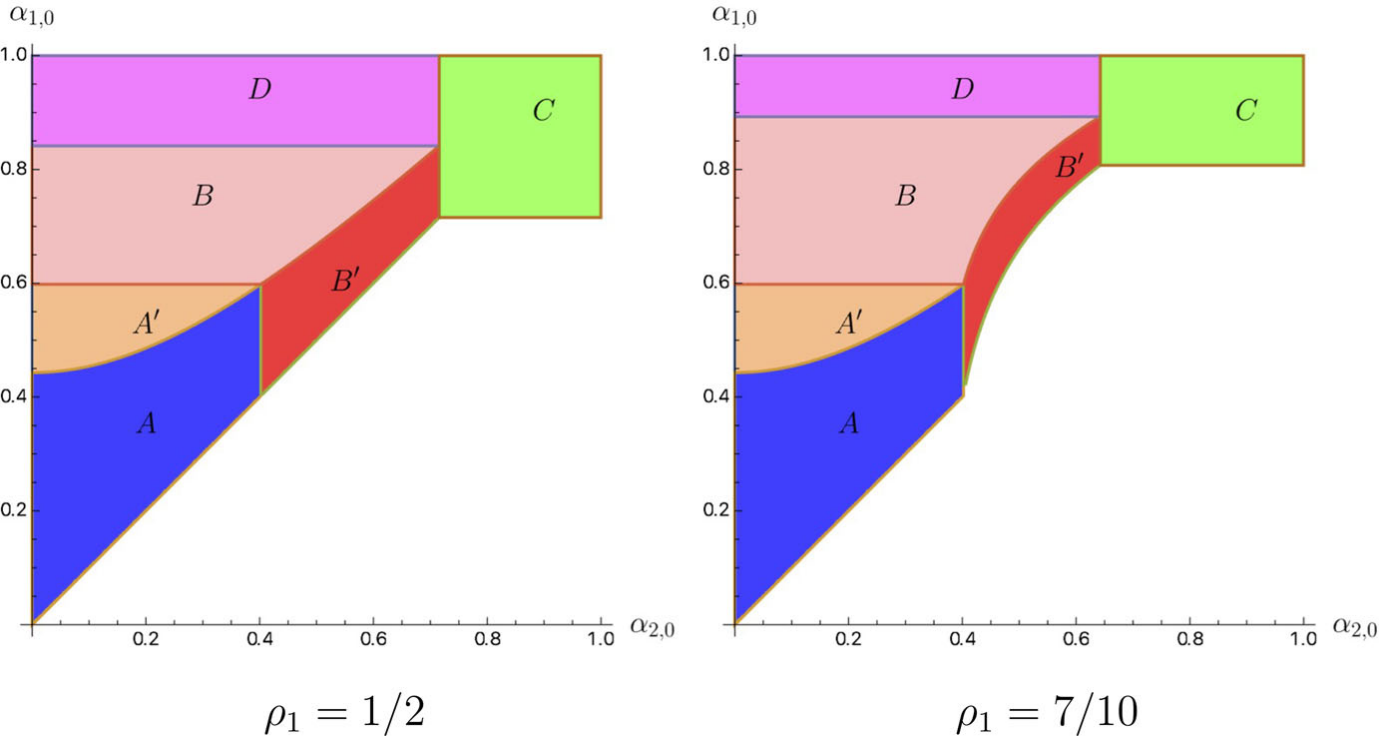
Shift of zones in response to $\rho _1$ increase

We prove this lemma in “Appendix B.3”. What Lemma 3 also says is that ${\underline{\alpha }}_1(\alpha _{2,0},\rho _1,\rho _2)$ and ${\underline{\alpha }}_1(\alpha _{2,0},\rho _1,\rho _2)+\frac{r^2\rho _2^2}{\alpha _{2,0}\rho _2^2+\rho _2\phi }$ are increasing in $\rho _1$. Figure 7 depicts how zones shift with respect to an increase in $\rho _1$.

It would also be helpful to study how *C* changes, varying the tie-breaking rule, and find the range of priors that *C* can span. From the definition of *C*, it is easy to see that with the increase of $\rho _1$, the lower bound on $\alpha _{1,0}$ increases, while the lower bound on $\alpha _{2,0}$ decreases. In order to determine the frontier of points spanned by *C*, consider the point $(\alpha _{1,0},\alpha _{2,0})=\left( \frac{1-r}{1-\rho _1r},\frac{1-r}{1-(1-\rho _1)r}\right)$. It lies at the intersection of *C*’s lower bounds on $\alpha _{1,0}$ and $\alpha _{2,0}$. Note that for any such point, it holds that

$$\begin{aligned} \frac{d\alpha _{1,0}}{d\alpha _{2,0}}=\frac{d\alpha _{1,0}/d\rho _1}{d\alpha _{2,0}/d\rho _1}=-\frac{(1-r)r/\left( 1-\rho _1r\right) ^2}{(1-r)r/\left( 1-\rho _2r\right) ^2}=-\frac{\alpha _{1,0}^2}{\alpha _{2,0}^2}. \end{aligned}$$

After solving the above differential equation with initial condition $\alpha _{1,0}\left( \frac{2-2r}{2-r}\right) =\frac{2-2r}{2-r}$, we get the expression for the frontier:

$$\begin{aligned} \alpha _{1,0}^{frontier}(\alpha _{2,0})=\frac{(1-r)\alpha _{2,0}}{(2-r)\alpha _{2,0}-(1-r)}. \end{aligned}$$

We can now state the result regarding *C*:

### Lemma 4

Zone *C* spans the region $\alpha _{1,0}\geqslant \frac{\alpha _{2,0}(1-r)}{\alpha _{2,0}(2-r)-(1-r)}$, $\alpha _{2,0}\in (1-r,1)$.

**Fig. 8:**
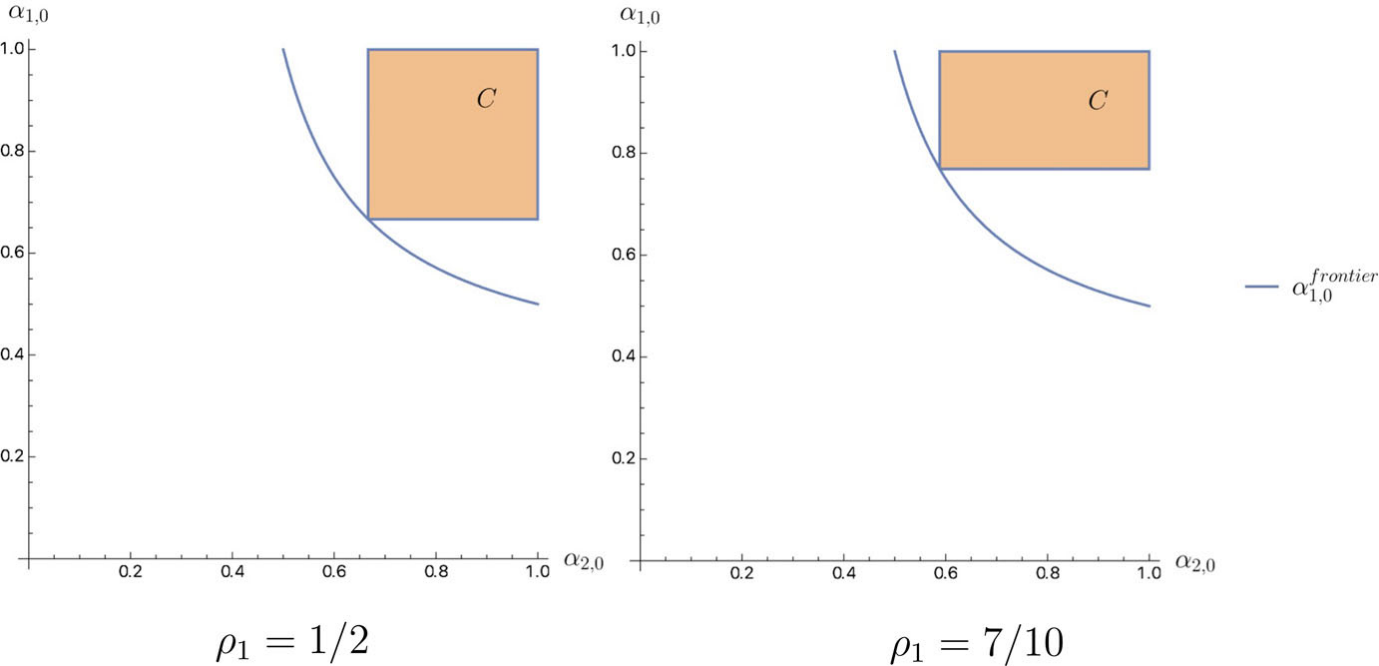
Shift of C in response to $\rho _1$ increase

Figure 8 depicts the effect of $\rho _1$ on *C* together with the $\alpha _{1,0}^{frontier}$.

### Investor-preferred tie-breaking rule

With the above comparative statics results at hand, let us analyze the tie-breaking rules that maximize the investor’s expected payoff. In this section, we focus on the case of $\alpha _{1,0}\geqslant \alpha _{2,0}$.^5^

Recall that the tie-breaking rule is a part of the equilibrium. *Before* choosing the experiments, entrepreneurs have to form the correct beliefs about the way the investor will break any ties *after* the results of the experiments are realized. How can the investor *choose* the tie-breaking probabilities? Without being too formal, we could allow the investor to make announcements about the intended tie-breaking before the entrepreneurs choose their experiments. Unlike the announcements in the form of “only projects with posterior equal to 1 will be funded,” statements about tie-breaking do not require the commitment to follow through. We can then focus on such equilibria in which entrepreneurs believe the investor and the investor makes truthful announcements.

Zone *C* allows for the investor’s first-best payoff. For priors exactly at the frontier $\alpha _{1,0}^{frontier}(\alpha _{2,0})$, there is a unique tie-breaking rule that induces the fully informative equilibrium. For the priors strictly above, there are multiple such rules. In the corollary below, we state the tie-breaking probabilities that implement full information and that are closest to the “fair” probabilities $(\rho _1,\rho _2)=(1/2,1/2)$ (also note that for priors in the square segment, $(\alpha _{1,0},\alpha _{2,0})\in \left[ \frac{2(1-r)}{2-r},1\right] ^2$ the “fair” rule already implements the fully informative equilibrium, so we do not specify a separate rule for that region):

#### Corollary 1

The region $\alpha _{1,0}\geqslant \frac{\alpha _{2,0}(1-r)}{\alpha _{2,0}(2-r)-(1-r)}$, $\alpha _{2,0}\in (1-r,1)$ allows for fully informative equilibrium under some tie-breaking rule. For $\alpha _{1,0}\geqslant \alpha _{2,0}$, $(\alpha _{1,0},\alpha _{2,0})\notin \left[ \frac{2(1-r)}{2-r},1\right] ^2$, one such tie-breaking rule is $\left( \rho _1,\rho _2\right) =\left( \frac{(1-r)(1-\alpha _{2,0})}{r\alpha _{2,0}},\frac{\alpha _{2,0}-(1-r)}{r\alpha _{2,0}}\right)$.

Call the set of priors for which the first-best investor’s payoff is achievable ${\overline{C}}$.

Let us now determine the preferred tie-breaking rule when the investor’s first-best is not achievable. We will focus on the case of $\alpha _{1,0}\geqslant \alpha _{2,0}$, with the remainder being symmetric. We can further exclude from the analysis the region $\left\{ \alpha _{1,0}\leqslant \frac{1+r^2}{2},\alpha _{2,0}\leqslant \frac{1-r^2}{2}\right\}$, because the distributions of posteriors there do not depend on $\rho$.

For the remaining zones, $B(\alpha _{1,0},\alpha _{2,0};\rho _1,\rho _2)$, $B^{\prime }(\alpha _{1,0},\alpha _{2,0};\rho _1,\rho _2)$, and $D(\alpha _{1,0},\alpha _{2,0};\rho _1,\rho _2)$, it is useful to define their respective “mirror” zones as:

$$\begin{aligned} B^{M}(\alpha _{1,0},\alpha _{2,0};\rho _1,\rho _2)&=B(\alpha _{2,0},\alpha _{1,0};\rho _2,\rho _1),\\ B^{\prime M}(\alpha _{1,0},\alpha _{2,0};\rho _1,\rho _2)&=B^{\prime }(\alpha _{2,0},\alpha _{1,0};\rho _2,\rho _1),\\ D^{M}(\alpha _{1,0},\alpha _{2,0};\rho _1,\rho _2)&=D(\alpha _{2,0},\alpha _{1,0};\rho _2,\rho _1). \end{aligned}$$

**Fig. 9:**
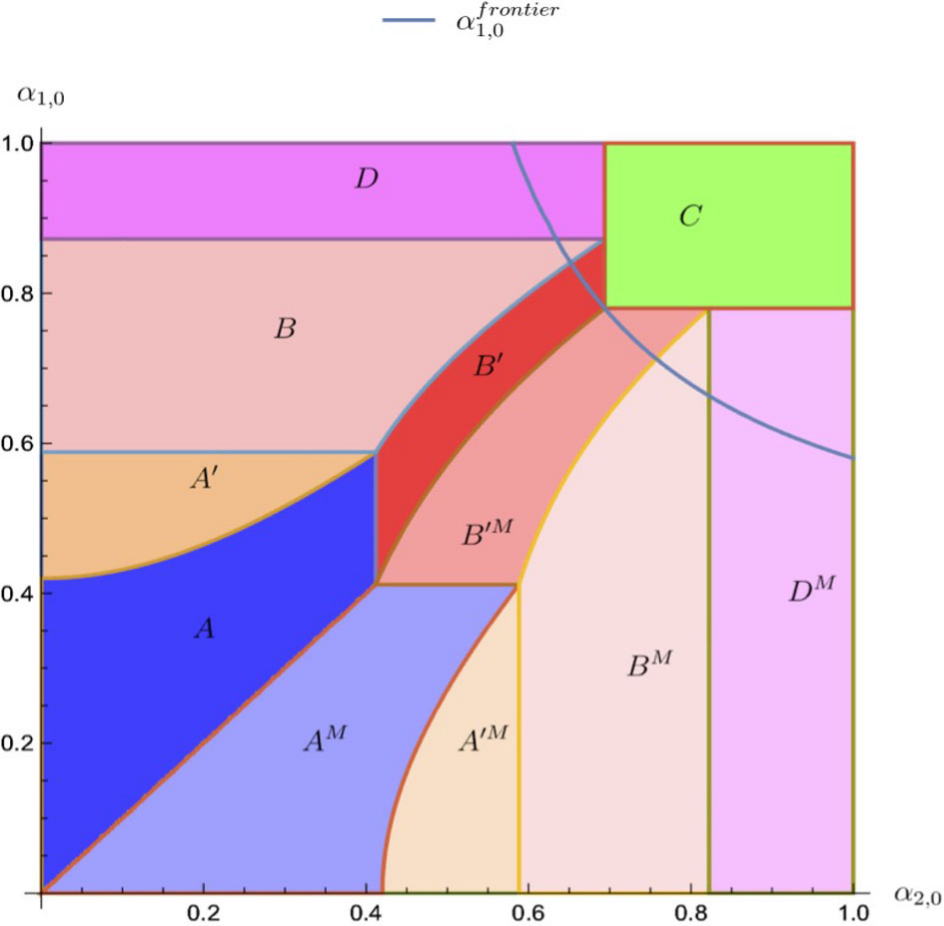
Zones with mirrors, $\rho _1=.39$, $r=.42$

Figure 9 depicts the zones together with their “mirror”—counterparts.

Regions $B^{\prime }$ and $B^{\prime M}$ are adjacent to each other. The regions border along the line ${\underline{\alpha }}_1$ (as in 3.5). The investor’s payoff increases with $\rho _1$ in $B^{\prime }$ but decreases in $B^{\prime M}$ because $\rho _1$ and $\rho _2$ switch roles. Besides, line ${\underline{\alpha }}_1$ is increasing in $\rho _1$. Thus, for a fixed prior $(\alpha _{1,0},\alpha _{2,0})$ initially in region $B^{\prime }$, the payoff increases with $\rho _1$ until the prior is at line ${\underline{\alpha }}_1$ and then decreases with $\rho _1$ when the prior moves to region $B^{\prime M}$. For every prior in $\left\{ \alpha _{1,0}\geqslant \max \{\frac{1-r^2}{2},\alpha _{2,0}\},\alpha _{2,0}\geqslant \frac{1-r^2}{2}\right\} \backslash {\overline{C}}$, define $(\rho ^{\star }_1,\rho ^{\star }_2)$ as a tie-breaking rule, which places this prior on the border between $B^{\prime }$ and $B^{\prime M}$, i.e.

$$\begin{aligned} \rho _1^{\star }(\alpha _{1,0},\alpha _{2,0})=\{\rho _1|\alpha _{1,0}={\underline{\alpha }}_1(\alpha _{2,0},\rho _1,1-\rho _1)\}. \end{aligned}$$

**Fig. 10:**
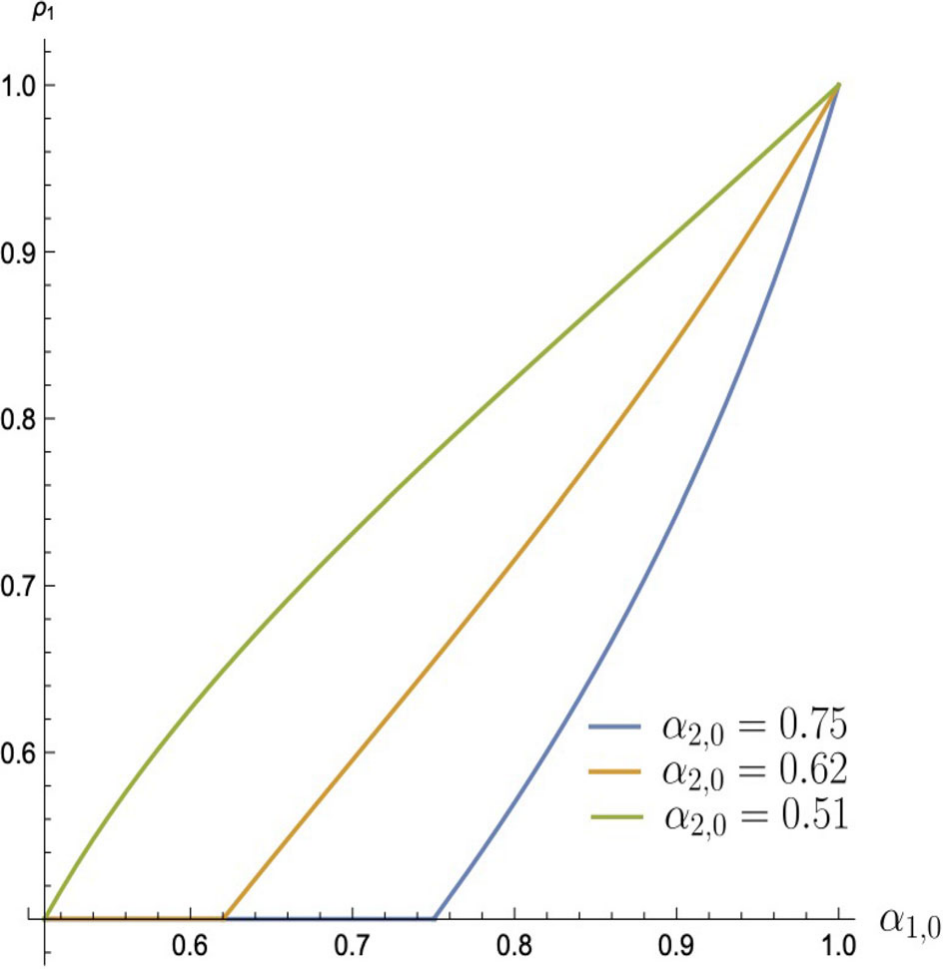
$\rho _1^{\star }(\alpha _{1,0},.)$ for various levels of $\alpha _{2,0}$

Naturally, such a tie-breaking rule allows the investor to achieve a local maximum. Figure 10 depicts the behavior of $\rho ^{\star }$ as a function of $\alpha _{1,0}$ for various levels of $\alpha _{2,0}$. The other two types of tie-breaking rules which can achieve local maxima are

``rule 0'' $$\begin{aligned} \rho _1\in {\left\{ \begin{array}{ll} \left[ 0,\frac{\alpha _{1,0}-(1-r+r^2)}{r(\alpha _{1,0}-r)}\right] , &{}\quad \text {if }\alpha _{1,0}\geqslant 1-r+r^2\\ \{0\},&{}\quad \text {if }\alpha _{1,0}<1-r+r^2. \end{array}\right. } \end{aligned}$$

and

``rule 1'' $$\begin{aligned} \rho _1\in {\left\{ \begin{array}{ll} \left[ \frac{(1-\alpha _{2,0})(1-r)}{r(\alpha _{2,0}-r)},1\right] , &{}\quad \text {if }\alpha _{2,0}\geqslant 1-r+r^2\\ \{1\},&{}\quad \text {if }\frac{1+r^2}{2}\leqslant \alpha _{2,0}<1-r+r^2. \end{array}\right. } \end{aligned}$$

“Rule 0” either moves a prior into zone *D* (for $\alpha _{1,0}\geqslant 1-r+r^2$) or into zone *B* with $\rho _1=0$ (for $\alpha _{1,0}<1-r+r^2$). In zone *B*, the investor’s payoff decreases with $\rho _1$, so setting it as low as possible within zone *B* is desirable. In “Appendix C”, we characterize an equilibrium in *B* under $\rho _1=0$. In zone *D*, the investor’s payoff is at the locally optimal level of $\alpha _{1,0}+\alpha _{2,0}-\alpha _{1,0}\alpha _{2,0}-r$.

Adopting the “rule 1” allows the investor to achieve the same payoff of $\alpha _{1,0}+\alpha _{2,0}-\alpha _{1,0}\alpha _{2,0}-r$ when the prior can move to zones $B^{M}$ or $D^M$ (i.e. when $\alpha _{2,0}\geqslant \frac{1+r^2}{2}$).

**Fig. 11:**
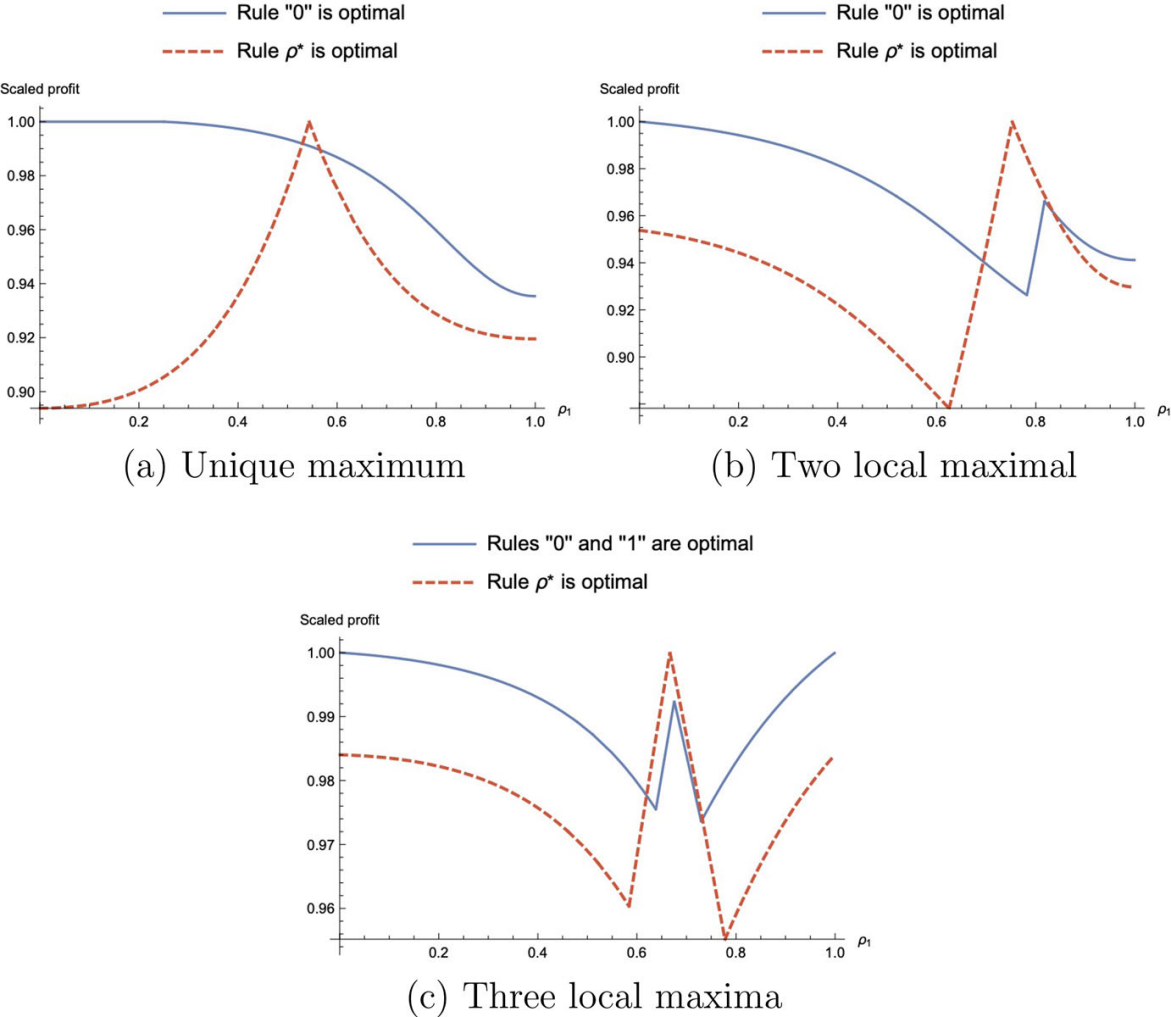
Investor’s payoff from $\rho _1$ under various parameters

We summarize these observations in the proposition below with the formal analysis in “Appendix D”.

#### Proposition 2

Consider the priors $\alpha _{1,0}\geqslant \alpha _{2,0}$ outside of zones $A(\alpha _{1,0},\alpha _{2,0})$, $A^{\prime }(\alpha _{1,0},\alpha _{2,0})$, and ${\overline{C}}$. If $\alpha _{1,0}\ne \alpha _{2,0}$, the investor benefits from a non-symmetric tie-breaking rule, $(\rho _1,\rho _2)\ne (1/2,1/2)$.

- If $\alpha _{2,0}<\frac{1-r^2}{2}$, the optimal tie-breaking rule is “rule 0”,$\rho _1\in [0,\max \{0,\frac{\alpha _{1,0}-(1-r+r^2)}{r(\alpha _{2,0}-r)}\}]$;
- If $(\alpha _{1,0},\alpha _{2,0})\in \left[ \frac{1-r^2}{2},\frac{1+r^2}{2}\right] ^2$, the optimal tie-breaking rule is $\rho _1^{\star }(\alpha _{1,0},\alpha _{2,0})$;
- If $(\alpha _{1,0},\alpha _{2,0})>(\frac{1+r^2}{2},\frac{1-r^2}{2})$, or $(\alpha _{1,0},\alpha _{2,0})>(\frac{1-r^2}{2},\frac{1+r^2}{2})$, $\rho _1^{\star }$ and “rule 0” achieve local maxima; which of them is globally optimal, depends on parameters;
- Moreover, if $\alpha _{2,0}>(1+r^2)/2$, “rule 1” also achieves a local maximum.

**Fig. 12:**
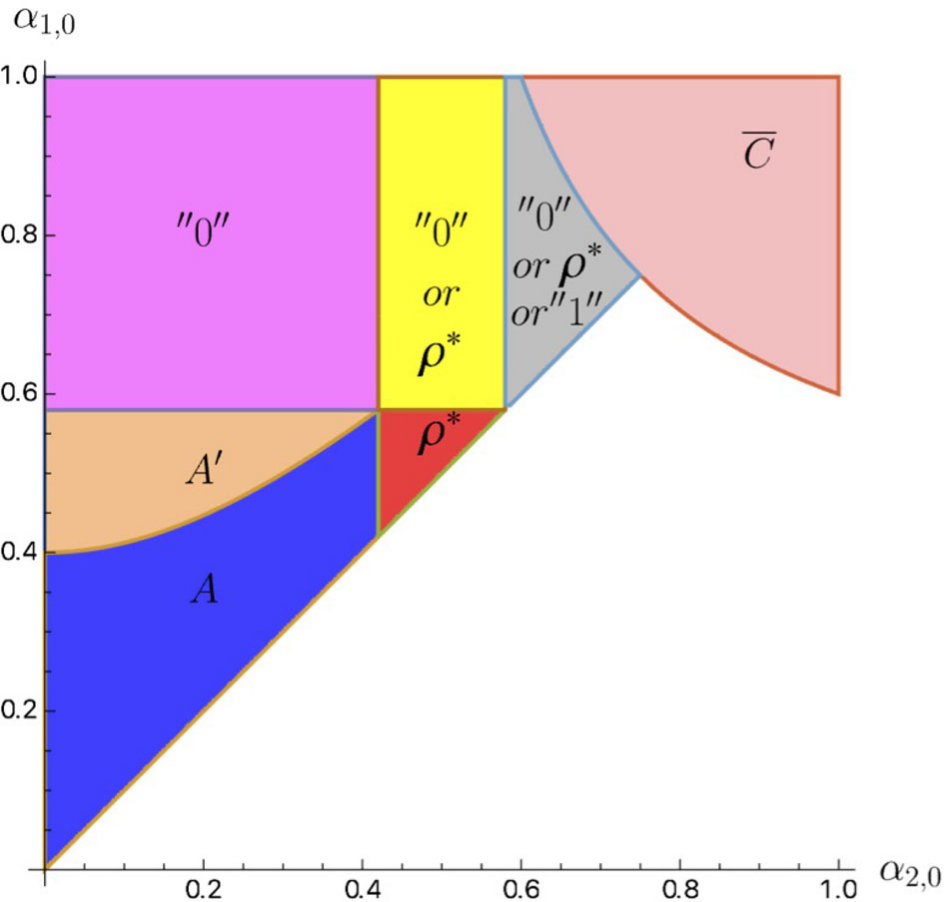
Zones with respect to optimal rules

We illustrate the results of Proposition 2 on Fig. 11 graphically, where we plot the investor’s profit normalized by the respective optimal level of profit. There, in panel (a), for the blue solid line, parameters are in region $\left[ \frac{1+r^2}{2},1\right) \times \left( 0,\frac{1-r^2}{2}\right]$; and for the red dashed line—in region $\left[ \frac{1-r^2}{2},\frac{1+r^2}{2}\right] ^2$; in panel (b), parameters are in region $\left[ \frac{1+r^2}{2},1\right) \times \left[ \frac{1-r^2}{2},\frac{1+r^2}{2}\right]$; and in panels (c)—in region $\left[ \frac{1+r^2}{2},1\right) \times \left[ \frac{1+r^2}{2},1\right)$. Furthermore, Fig. 12 shows which of the tie-breaking rules achieve local maximum depending on the parameter region.

Finally, let us establish the conditions for which the investor prefers the internal tie-breaking rule $\rho ^{\star }$ to either of the “corner” rules. For every pair of priors in $[\frac{1-r^2}{2},1)^2\backslash {\overline{C}}$, there is $\rho ^{\star }$ that places the prior on the border between $B^{\prime }$ and $B^{\prime M}$. Note also that under $\rho ^{\star }$, both entrepreneurs have atoms at $\{0\}\cup \{1\}$, continuously distributed mass of posteriors in $(r,{\overline{\alpha }})$, but no atoms at *r*. If we fix an initial prior $(\alpha _{1,0},\alpha _{2,0})$ and corresponding $\rho ^{\star }(\alpha _{1,0},\alpha _{2,0})$, there exists a path along ${\underline{\alpha }}_1(\alpha _{2,0},\rho _1^{\star },1-\rho _1^{\star })$ towards the first-best region ${\overline{C}}$. Along that path, the distributions of posteriors are ordered in the sense of *First Order Stochastic Dominance*: as $\alpha _{2,0}$ increases, the equilibrium distributions increase. As priors get sufficiently close to ${\overline{C}}$, the continuous parts of both distributions shrink, and the distributions approach the perfectly informative distributions,

$$\begin{aligned} {\hat{\alpha }}_i= {\left\{ \begin{array}{ll} 1,&{}\quad \text { w.p. }\alpha _{i,0},\\ 0,&{}\quad \text { w.p. }1-\alpha _{i,0}, \end{array}\right. } \end{aligned}$$

and the investor’s payoff approaches the first-best payoff $(\alpha _{1,0}+\alpha _{2,0}-\alpha _{1,0}\alpha _{2,0})(1-r)$.

On the other hand, for priors we consider under “0 rule” and “1 rule”, the payoff remains fixed at $\alpha _{1,0}+\alpha _{2,0}-\alpha _{1,0}\alpha _{2,0}-r$. The latter payoff is strictly lower than the first-best payoff. There exist priors sufficiently close to ${\overline{C}}$, such that the distributions of posteriors under $(\rho ^{\star }_{1},1-\rho ^{\star }_{1})$ are sufficiently close to the perfectly informative distributions, and thus, the investor’s profit is sufficiently close to the first-best payoff and is also higher than $\alpha _{1,0}+\alpha _{2,0}-\alpha _{1,0}\alpha _{2,0}-r$. We can conclude that for priors close to ${\overline{C}}$, the investor prefers the internal tie-breaking rule $(\rho _1^{\star },1-\rho _1^{\star })$.

#### Corollary 2

If the pair of priors is close enough to ${\overline{C}}$, the investor prefers the tie-breaking rule $(\rho _1^{\star },1-\rho _1^{\star })$ to the corner “0 rule”.

It is worthwhile to mention that the investor might prefer the corner “0 rule” for robustness reasons: unlike the $\rho ^{\star }$-rule, “0 rule” does not require the exact knowledge of priors, but only that priors are in a certain range, $\alpha _{1,0}\geqslant \max \{\frac{1+r^2}{2},\alpha _{2,0}\}$, $(\alpha _{1,0},\alpha _{2,0})\notin {\overline{C}}$. The “0 rule” might also be more attractive from the fairness perspective: it discriminates against the initially stronger entrepreneur 1, whereas the $\rho ^{\star }$-rule discriminates against the weaker entrepreneur 2.

One more interesting observation is that the symmetry of priors does not imply the global optimality of the symmetric tie-breaking rule. To see this, consider a numeric example: if $\alpha _{1,0}=\alpha _{2,0}=0.6$, $r=0.3$, the profit from the “0”-rule is 0.54 while the profit from the fair tie-breaking is 0.536. Notice also that if we increase the priors to $\alpha _{1,0}=\alpha _{2,0}=0.7$, the order switches, and the profits become 0.61 and 0.617, respectively.

## Discussion

The approach studied in this paper allows characterizing equilibria with arbitrary tie-breaking rules, which is a generalization of the analysis in Boleslavsky and Cotton (2018). There, they study an equilibrium with the symmetric tie-breaking rule only. Leveraging the generality of the analysis, we also extend the analysis to find the investor-preferred tie-breaking rule. Using the methods from Boleslavsky and Cotton (2018) to study a general tie-breaking rule would reduce tractability. To see this, note that with a symmetric tie-breaking rule, the expressions in Subsection 3.2. significantly simplify. Under $\rho _1=\rho _2=1/2$, the bounds of $B^{\prime }$ on $\alpha _{1,0}$ become $\alpha _{1,0}\in \left[ \alpha _{2,0},\sqrt{\alpha _{2,0}^2+r^2}\right]$, coinciding with the bounds on $\alpha _{1,0}$ in $A^{\prime }$. Similarly, the bounds of *B* on $\alpha _{2,0}$ reduce to $\alpha _{2,0}\in \left( 0,\sqrt{\alpha _{1,0}^2-r^2}\right)$, as in zone *A*. Moreover, the expressions for values $(V_1,V_2)$ in zones *B* and $B^{\prime }$ become equal to those in zones *A* and $A^{\prime }$, respectively. Hence, it is a manageable task to write down equilibrium distributions in terms of priors in zones *B* and $B^{\prime }$ in the case of $\rho _1=1/2$ (unlike in the case of general $\rho _1 \in (0,1)$).

The equilibrium analysis under the symmetric tie-breaking rule, $\rho _1=\rho _2=1/2$ in Boleslavsky and Cotton (2018), relies on the direct analysis of the Lagrangian as in expressions (*L*)–(${\tilde{L}}$). After proving some general properties of equilibrium distributions (uniformly distributed continuous part and atoms only at 0, *r*, and 1), the analysis there proceeds with establishing the supports of strategies and zones where different types of equilibria hold. A set of conditions necessary for equilibrium are used to identify the latter. In the case of symmetric tie-breaking, the expressions that pin down the supports and equilibrium regimes are tractable. However, allowing for a general tie-breaking would require working with algebraically more complex expressions, especially in the zones where there are atoms at the posterior 1.

Another complexity that our approach simplifies is the comparative statics of distributions. With general tie-breaking, the expressions of equilibrium CDFs in terms of priors and $(\rho _1,\rho _2)$ feature many non-linear terms. They do not allow making unambiguous conclusions about the effect of parameters’ changes.

However, writing the expressions in terms of auxiliary values is straightforward and allows for tractable analysis.

Our approach can also be applied to the information design problem with three senders. In Muratov (2022), we characterize equilibria in the three-player all-pay auction with a reserve and a bid cap (being set at 1), building on the work of Baye et al. (1996). We show that there exists a non-degenerate region of valuations $V_1\geqslant V_2=V_3>1$, in which the supports of the three players’ strategies take the following form: player 1: $\{0\}\cup [r,{\overline{b}})\cup \{1\}$ (with mass at 0 only if $V_1=V_2=V_3$); player 2: $\{0\}\cup (r,{\overline{b}})\cup \{1\}$; player 3: $\{0\}\cup ({\underline{b}},{\overline{b}})\cup \{1\}$. Here, ${\overline{b}}\in (r,1)$ and the identities of players with equal valuations are up to a permutation. If three players are actively bidding between *r* and 1, the third player can join the continuous bidding anywhere between *r* and ${\overline{b}}$. If there is continuous bidding, distributions are such that any single active player faces a uniform distribution of the maximum of competitors’ bids. Knowledge of this equilibrium structure can be used to characterize corresponding equilibria in the three–sender information design game.

In a related paper, Szech (2015) studies the role of tie-breaking rules in the all-pay auction with bid caps, which generalizes the analysis of Che and Gale (1998). After establishing the equilibrium under the general bid cap and tie-breaking rule, Szech (2015) studies the principal’s design problem, whose goal is to maximize the expected sum of bids. She concludes that it is optimal to favor the weaker bidder and, if the choice is among the simple tie-breaking rules, to do so deterministically. That is, to always award the prize to the weak player in case of a tie. Our analysis focuses on tie-breaking rules in the information design environment, where the principal prefers higher informativeness under fixed average total expenditures. Similarly to Szech (2015), always breaking the tie in favor of the weaker player can be optimal, but not generally. Moreover, breaking the tie in favor of the stronger player with a higher probability can outperform the latter rule.

For some intuition why favoring a stronger player can sometimes be beneficial, consider a numeric example. Let $r=0.3$, $\alpha _{1,0}=0.89$, $\alpha _{2,0}=0.81$. Under fair tie-breaking, the priors are in zone *D*. In equilibrium, player 1’s realized posterior is 0.3 with probability 11/70 and 1 with probability 59/70, while player 2 chooses full information (1 w.p. 0.81, 0 w.p. 0.19). Switching to full information for player 1 has a gain of a higher chance of a unit posterior and a loss from not having a posterior realization of $r=0.3$. Player 1 does not want to risk switching to full information because the gain from it will be diluted by the fact that the prize will be fairly shared with player 2 in case of a tie. To motivate this switch for player 1, he has to be promised a higher chance of obtaining the prize in case of a tie at posterior 1. Thus, tie-breaking probabilities $(\rho _1,\rho _2)=(0.55,0.45)$, for instance, support the full information by both players. Similar reasoning applies in zone $B^{\prime }$.

In this article, we employ the knowledge of contests to better understand an information design game. A growing literature also combines the two disciplines, focusing on applying information design in contests. For example, there are studies of optimal principal’s disclosure policy in Tullock contests (Zhang and Zhou 2016), all-pay auctions (Chen et al. 2017), and binary action contests (Ponce 2018). Besides the player ability-types, articles also study disclosing information about the number of contestants (Feng and Lu 2016) and Tullock discrimination parameter (Feng 2020).

## Conclusion

In this paper we have formally established and characterized the mapping between a class of information design games and a class of all-pay auctions. We have shown that solving for the equilibria in the all-pay auction is helpful for finding the equilibria in the information design game. Building on the work of Boleslavsky and Cotton (2018), our approach allows for a tractable generalization to an arbitrary tie-breaking rule. Our approach also allows for straightforward comparative statics. We apply it to study the effects of tie-breaking rule on equilibrium informativeness and to determine the principal-preferred tie-breaking rule. With the help of our approach, we can conclude that the investor benefits from a non-symmetric tie-breaking rule. We further find that there can be up to three distinct types of tie-breaking rules that are locally optimal. Under some circumstances, breaking the tie in favor of the weaker sender can be optimal. However, favoring the stronger sender can also be optimal in some other cases.

In general, the approach we suggest in this paper can potentially be extended to study mappings between other classes of information design games and corresponding contests. Characterizing these mappings would be useful to study both types of games, and we leave that for future research.

## Deriving the correspondences

As we go through the regions of valuations $(V_1,V_2)$ outlined in 3.2, we state the distributions, find the expressions for averages, and then derive the mappings from averages of expenditures to valuations, inverting the expressions.

*Region* *A* Following the derivations in Muratov (2022), in region *A*, bids are distributed according to the CDFs:

$$\begin{aligned} G_1(x)= {\left\{ \begin{array}{ll} 0, &{}\quad \text { if } x<r\\ \frac{x}{V_2}, &{}\quad \text { if } x\in [r,V_2)\\ 1, &{}\quad \text { if } x\geqslant V_2; \end{array}\right. }\quad G_2(x)= {\left\{ \begin{array}{ll} \frac{r+V_1-V_2}{V_1}, &{}\quad \text { if } x<r\\ \frac{x+V_1-V_2}{V_1}, &{}\quad \text { if } x\in [r,V_2)\\ 1, &{}\quad \text { if } x\geqslant V_2. \end{array}\right. } \end{aligned}$$

The pair of expenditures is then

$$\begin{aligned} \alpha _{1,0}&=\frac{r^2}{V_2}+\int _{r}^{V_2}\frac{x}{V_2}dx=\frac{r^2+V_2^2}{2V_2},\; \alpha _{2,0}=\frac{V_2^2-r^2}{2V_1}. \end{aligned}$$

Solving for the valuations $(V_1,V_2)$ and selecting the solution such that $V_1\geqslant 0$ yields the expressions in 3.9. Using the fact that in the region *A*, $V_2\in [r,1)$ and $V_1\in (V_2,+\infty )$, and the expressions above, we can translate the boundaries of region *A* in terms of valuations into the terms of expenditures, obtaining the expression in 3.1.

*Region* $A^{\prime }$ For the case $V_2=V_1\leqslant 1$, there are two classes of equilibria: those where player 1 can have an atom at *r*, and those where player 2 can have an atom at *r*. In every equilibrium the bids are distributed according to the CDFs:

$$\begin{aligned} G_1(x)= {\left\{ \begin{array}{ll} \frac{r(1-t)}{V_2}, &{}\quad \text { if } x<r\\ \frac{x}{V_2}, &{}\quad \text { if } x\in [r,V_2)\\ 1, &{}\quad \text { if } x\geqslant V_2; \end{array}\right. }\quad G_2(x)= {\left\{ \begin{array}{ll} \frac{r(1-q)}{V_2}, &{}\quad \text { if } x<r\\ \frac{x}{V_2}, &{}\quad \text { if } x\in [r,V_2)\\ 1, &{}\quad \text { if } x\geqslant V_2; \end{array}\right. } \end{aligned}$$

where *t* (*q*) is a parameter such that $\frac{t\times r}{V_2}$ $\left( \frac{q\times r}{V_2}\right)$ is the size of the atom that player 1 (2) has at *r*, and $t\in [0,1]$, $q\in [0,1]$, $t\times q=0$. Throughout the work we focus on equilibria with $q=0$, since we only need to describe the outcomes with expenditures of player 1 being above the expenditures of player 2, with the remainder of cases being symmetric.

The expenditures under $q=0$ are:

$$\begin{aligned} \alpha _{1,0}&=\frac{r^2\times t}{V_2}+\int _{r}^{V_2}\frac{x}{V_2}dx=\frac{V_2^2-r^2(1-2t)}{2V_2},\;\alpha _{2,0}=\frac{V_2^2-r^2}{2V_2}. \end{aligned}$$

Solving for $V_2$ in terms of $\alpha _{2,0}$ and also in terms of $(\alpha _{1,0},t)$, and selecting such solution that $V_2>0$, we have

$$\begin{aligned} V_2=\alpha _{2,0}+\sqrt{\alpha _{2,0}^2+r^2}=\alpha _{1,0}+\sqrt{\alpha _{1,0}+r^2(1-2t)}. \end{aligned}$$

Varying $V_2$ from *r* to 1, and *t* from 0 to 1, we obtain the expression 3.2.

*Region* *B* If $1<V_2\leqslant \frac{1-\rho _1 r}{\rho _2}$, $\rho _2^2V_2-\rho _2<\rho _1^2V_1-\rho _1$, in the unique equilibrium of the all-pay auction game, the players’ bids are distributed according to the CDFs:

$$\begin{aligned} G_1(x)= {\left\{ \begin{array}{ll} 0, &{}\quad \text { if } x<r\\ \frac{x}{V_2}, &{}\quad \text { if } x\in \left[ r,{\tilde{x}}\right) \\ \frac{1-\rho _2V_2}{\rho _1V_2}, &{}\quad \text { if } x\in \left[ {\tilde{x}},1\right) \\ 1, &{}\quad \text { if } x\geqslant 1; \end{array}\right. }\quad G_2(x)= {\left\{ \begin{array}{ll} \frac{r+U^{\star }}{V_1}, &{}\quad \text { if } x<r\\ \frac{x+U^{\star }}{V_1}, &{}\quad \text { if } x\in \left[ r,{\tilde{x}}\right) \\ \frac{1-\rho _2V_2+\rho _1U^{\star }}{\rho _1V_1}&{}\quad \text { if } x\in \left[ {\tilde{x}},1\right) \\ 1, &{}\quad \text { if } x\geqslant 1, \end{array}\right. } \end{aligned}$$

where ${\tilde{x}}=\frac{1}{\rho _1}-\frac{\rho _2}{\rho _1}V_2$ and $U^{\star }=\frac{\rho _2-\rho _1+\rho _1^2V_1-\rho _2^2V_2}{\rho _1^2}>0$ (corresponds to the equilibrium payoff of player 1 in the all-pay auction game).

The average expenditures are $\alpha _{1,0}=\frac{r^2}{V_2}+\int _{r}^{{\tilde{x}}}\frac{x}{V_2}dx+\left( 1-\frac{1-\rho _2V_2}{\rho _1V_2}\right)$, $\alpha _{2,0}=\int _{r}^{{\tilde{x}}}\frac{x}{V_1}dx+\left( 1-\frac{1-\rho _2V_2+\rho _1U^{\star }}{\rho _1V_1}\right)$, or

A.1 $$\begin{aligned} \alpha _{1,0}&=\frac{(\rho _2-\rho _1)(V_2-1)^2+\rho _1^2(r^2+V_2^2)}{2\rho _1^2V_2}, \end{aligned}$$

A.2 $$\begin{aligned} \alpha _{2,0}&=\frac{\left( \rho ^2_2V_2^2-1\right) +\rho _1(2-\rho _1r^2)}{2\rho _1^2V_1}. \end{aligned}$$

Solving for valuations in terms of expenditures, and selecting the solution such that $1\leqslant V_2\leqslant \frac{1-\rho _1r}{\rho _2}$, yields the expressions in 3.11. Varying $V_2$ from 1 to $\frac{1-\rho _1r}{\rho _2}$ and $V_1$ from $\frac{\rho _2^2V_2-\rho _2+\rho _1}{\rho _1^2}$ to plus infinity we get that in terms of expenditures, the region is translated into $B(\alpha _{1,0},\alpha _{2,0};\rho _1,\rho _2)$, as in 3.3.

*Region* $B^{\prime }$ If $1<V_2\leqslant \frac{1-\rho _1 r}{\rho _2}$, $\rho _2^2V_2-\rho _2=\rho _1^2V_1-\rho _1$, there are multiple equilibria in the all-pay auction. In every equilibrium the players’ bids are distributed according to the CDFs:

$$\begin{aligned} G_1(x)= {\left\{ \begin{array}{ll} \frac{r(1-t)}{V_2}, &{}\quad \text { if } x<r\\ \frac{x}{V_2}, &{}\quad \text { if } x\in \left[ r,{\tilde{x}}\right) \\ \frac{1-\rho _2V_2}{\rho _1V_2}, &{}\quad \text { if } x\in \left[ {\tilde{x}},1\right) \\ 1, &{}\quad \text { if } x\geqslant 1; \end{array}\right. } \quad G_2(x)= {\left\{ \begin{array}{ll} \frac{r(1-q)}{V_1}, &{}\quad \text { if } x<r\\ \frac{x}{V_1}, &{}\quad \text { if } x\in \left[ r,{\tilde{x}}\right) \\ \frac{1-\rho _2V_2}{\rho _1V_1}&{}\quad \text { if } x\in \left[ {\tilde{x}},1\right) \\ 1, &{}\quad \text { if } x\geqslant 1, \end{array}\right. } \end{aligned}$$

where ${\tilde{x}}=\frac{1}{\rho _1}-\frac{\rho _2}{\rho _1}V_2$, and $r\in [0,1]$, $q\in [0,1]$, $t\times q=0$. Again, we focus on $q=0$.

The pair of expenditures is then

A.3 $$\begin{aligned} \alpha _{1,0}&=\frac{(V_2-1)^2(\rho _2-\rho _1)+\rho _1^2(r^2(2t-1)+V_2^2)}{2V_2\rho _1^2}, \end{aligned}$$

A.4 $$\begin{aligned} \alpha _{2,0}&=\frac{\rho _2^2V_2^2+\rho _1(2-r^2\rho _1)-1}{2(\rho _2^2V_2+\rho _1-\rho _2)}. \end{aligned}$$

Solving for $V_2$ and selecting the solution that falls within the proper range for $V_2$, we get two expressions for $V_2$, in terms of $(\alpha _{1,0},t)$ and $\alpha _{2,0}$:

$$\begin{aligned} V_2&=\frac{\rho _1(\alpha _{1,0}\rho _1-2)+\sqrt{\rho _2^2(\rho _1-\rho _2+r^2\rho _1^2(1-2t))+(1-2\rho _1+\alpha _{1,0}\rho _1^2)^2}}{\rho _2^2}\\&=\frac{\alpha _{2,0}\rho _2^2+\sqrt{\rho _2^2(\rho _2-\rho _1+r^2\rho _1^2-2\alpha _{2,0}(\rho _2-\rho _1))+(\alpha _{2,0}\rho _2^2)^2}}{\rho _2^2}. \end{aligned}$$

Varying $V_2$ from 1 to $\frac{1-\rho _1r}{1-\rho _1}$, and *t* from 0 to 1, we obtain the expressions 3.4 and 3.5.

*Region* *C* If $(V_1,V_2)\in \left[ \frac{1-r\rho _2}{\rho _1},\frac{1}{\rho _1}\right] \times \left[ \frac{1-r\rho _1}{\rho _2},\frac{1}{\rho _2}\right]$, there is an equilibrium in which the players bid according to

$$\begin{aligned} \text {bid}_1={\left\{ \begin{array}{ll} 0, &{}\quad \text { with probability } \frac{1-\rho _2V_2}{\rho _1 V_2},\\ 1, &{}\quad \text { with probability } \frac{V_2-1}{\rho _1 V_2}; \end{array}\right. }\quad \text {bid}_2={\left\{ \begin{array}{ll} 0, &{}\quad \text { with probability } \frac{1-\rho _1 V_1}{\rho _2V_1},\\ 1, &{}\quad \text { with probability } \frac{V_1-1}{\rho _2V_1}; \end{array}\right. } \end{aligned}$$

Then the average expenditures are $\alpha _{1,0}=(V_2-1)/(\rho _1V_2)$, $\alpha _{2,0}=(V_1-1)/(\rho _2V_1)$, and the valuations in terms of expenditures are $V_2=\frac{1}{1-\rho _1\alpha _{1,0}}$, $V_1=\frac{1}{1-\rho _2\alpha _{2,0}}$. Varying $V_1$ from $\frac{1-r\rho _2}{\rho _1}$ to $\frac{1}{\rho _1}$ and $V_2$ from $\frac{1-r\rho _1}{\rho _2}$ to $\frac{1}{\rho _2}$, we get expression 3.6.

*Region* $C^{\prime }$ If $(V_1,V_2)\in \left\{ \frac{1-\rho _2r}{\rho _1}\right\} \times \left[ \frac{1-r\rho _1}{\rho _2},\frac{1}{\rho _2}\right)$, there is a class of equilibria in which the players bid according to

$$\begin{aligned} \text {bid}_1={\left\{ \begin{array}{ll} 0, &{}\quad \text { with probability } \frac{(1-t)(1-\rho _2V_2)}{\rho _1V_2},\\ r, &{}\quad \text { with probability } \frac{t(1-\rho _2V_2)}{\rho _1V_2},\\ 1, &{}\quad \text { with probability } \frac{V_2-1}{\rho _1 V_2}; \end{array}\right. } \quad \text {bid}_2={\left\{ \begin{array}{ll} 0, &{}\quad \text { with probability } \frac{1-\rho _1 V_1}{\rho _2V_1}\\ 1, &{}\quad \text { with probability } \frac{V_1-1}{\rho _2V_1}; \end{array}\right. } \end{aligned}$$

where $t\in [0,1]$ is a free parameter. This translates into expenditures

$$\begin{aligned} \alpha _{1,0}=\frac{V_2-1-r\times t\left( \rho _2V_2-1\right) }{\rho _1V_2},\alpha _{2,0}=\frac{V_1-1}{\rho _2V_1}, \end{aligned}$$

and valuations in terms of priors

$$\begin{aligned} V_2=\frac{1-r\times t}{1-\rho _2 r\times t-\rho _1\alpha _{1,0}},V_1=\frac{1}{1-\rho _2\alpha _{2,0}}. \end{aligned}$$

Since $V_1=\frac{1-\rho _2r}{\rho _1}$ and varying $V_2$ from $\frac{1-r\rho _1}{\rho _2}$ to $\frac{1}{\rho _2}$ and *t* from 0 to 1, we get expression 3.7.

*Region* *D* If $(V_1,V_2)\in \left[ \frac{1-\rho _2 r}{\rho _1},\infty \right) \times \left[ \frac{1-\rho _1 r}{\rho _2},\frac{1}{\rho _2}\right]$, (region D), there is an equilibrium, in which the players bid according to

$$\begin{aligned} \text {bid}_1={\left\{ \begin{array}{ll} r, &{}\quad \text { with probability } \frac{1-\rho _2V_2}{\rho _1 V_2},\\ 1, &{}\quad \text { with probability } \frac{V_2-1}{\rho _1 V_2} ; \end{array}\right. } \quad \text {bid}_2={\left\{ \begin{array}{ll} 0, &{}\quad \text { with probability } \frac{V_1\rho _1-(1-r)}{V_1\rho _1},\\ 1, &{}\quad \text { with probability } \frac{1-r}{\rho _1 V_1}. \end{array}\right. } \end{aligned}$$

The expenditures are $\alpha _{1,0}=\frac{V_2(1-\rho _2r)-(1-r)}{\rho _1V_2}$, $\alpha _{2,0}=\frac{1-r}{\rho _1V_1},$ and valuations in terms of priors are $V_2=\frac{1-r}{1-\rho _1\alpha _{1,0}-\rho _2r}$, $V_1=\frac{1-r}{\rho _1\alpha _{2,0}}.$ As we vary $V_1$ and $V_2$, we obtain expression 3.8.

## Comparative statics

### Proof of Lemma 1

#### Proof

Consider the system of Eqs. A.1–A.2 that defines the expenditures in region *B*, and transform it to

$$\begin{aligned}&2\alpha _{1,0}V_2\rho _1^2-(1-2\rho _1)(V_2-1)^2-\rho _1^2(r^2+V_2^2)=0\\&2\alpha _{2,0}V_1\rho _1^2-(1-\rho _1)^2V_2^2-\rho _1(2-\rho _1r^2)+1=0, \end{aligned}$$

where we also use the fact that $\rho _1=1-\rho _2$. Call this system $\pmb {\Xi }$. Keeping the pair of expected bids/priors fixed, we treat $V_1$ and $V_2$ as implicit functions of $\rho _1$, that follow from $\pmb {\Xi }$. We are interested in the signs of $\frac{dV_1}{d\rho _1}$ and $\frac{dV_2}{d\rho _1}$. Denote the matrix of derivatives of $\pmb {\Xi }$ with respect to $\pmb {V}=(V_1,V_2)^T$ as *J*. Then, we have

$$\begin{aligned} J&=\begin{pmatrix} \frac{\partial \Xi _1}{\partial V_1} &{} \frac{\partial \Xi _1}{\partial V_2} \\ \frac{\partial \Xi _2}{\partial V_1} &{} \frac{\partial \Xi _2}{\partial V_2} \end{pmatrix}=\begin{pmatrix} 0 &{} 2\left( 1-V_2(1-\rho _1)^2-\rho _1(2-\alpha _{1,0}\rho _1) \right) \\ 2\alpha _{2,0}\rho _1^2 &{} -2(1-\rho _1)^2V_2 \end{pmatrix} . \end{aligned}$$

Keeping in mind that $d\rho _2=-d\rho _1$, the vector of derivatives of $\pmb {\Xi }$ with respect to $\rho _1$ is

$$\begin{aligned} \begin{pmatrix} \frac{\partial \Xi _1}{\partial \rho _1}\\ \frac{\partial \Xi _2}{\partial \rho _1} \end{pmatrix}&= \begin{pmatrix} 2\left( (V_2-1)^2-\rho _1(r^2+V_2^2)+2V_2\alpha _{1,0}\rho _1\right) \\ 2\left( V_2^2(1-\rho _1)+r^2\rho _1+2V_1\alpha _2\rho _1-1\right) \end{pmatrix}. \end{aligned}$$

By Implicit Function Theorem, $\frac{d\; \pmb {V}}{d \;\rho _1}=-J^{-1}\times \frac{\partial \; \pmb {\Xi }}{\partial \; \rho _1}$. After plugging in the values for $(\alpha _{1,0},\alpha _{2,0})$ from (A.1)–(A.2) and some simplifications, we get that

$$\begin{aligned} \frac{dV_1}{d\rho _1}&=\frac{2\rho _2V_1(V_2-1)\left( (1+V_2)(1-\rho _1(2-r^2\rho _1))-\rho _2^2V_2^2(3-V_2)\right) }{\rho _1\left( V_2^2\rho _2^2+\rho _1\left( 2-r^2\rho _1\right) -1\right) ^2},\\ \frac{dV_2}{d\rho _1}&=\frac{2(V_2-1)^2V_2\rho _2}{\rho _1\left( \rho _2^2V_2^2+\rho _1(2-r^2\rho _1)-1\right) }. \end{aligned}$$

To determine the sign of $\frac{dV_1}{d\rho _1}$ first, note that the denominator is always positive. Second, the numerator is always non-positive, since the term $\gamma (V_2)\doteq (1+V_2)(1-\rho _1(2-r^2\rho _1))-\rho _2^2V_2^2(3-V_2)$ is negative: as a function of $V_2$ it achieves a local maximum (minimum) at $V_2=1-\frac{\sqrt{2-\rho _1(4-\rho _1(3-r^2))}}{\sqrt{3}\rho _2}<1$ ($V_2=1+\frac{\sqrt{2-\rho _1(4-\rho _1(3-r^2))}}{\sqrt{3}\rho _2}>1$). Plugging the two extreme values ($V_2\in \{1,\frac{1-\rho _1r}{1-\rho _1}\}$) results in both values of $\gamma (V_2)$ being negative. It shows that $\gamma (V_2)$ is indeed negative. Overall, $\frac{dV_1}{d\rho _1}$ is negative in *B* and strictly so for $V_2>1$.

As for the sign of $\frac{dV_2}{d\rho _1}$, its numerator is always positive. The denominator is increasing in $V_2$ and attains the smallest value of $(1-r^2)\rho _1^2>0$ at $V_2=1$. Hence, $\frac{dV_2}{d\rho _1}\geqslant 0$ in the *B*–zone.

We can now make conclusions about the stochastic ordering of distributions of posteriors with respect to the tie-breaking rule. The slope of $G_1\left( {\hat{\alpha }}\right)$ in the increasing part is $1/V_2$. With an increase of $\rho _1$, $V_2$ increases and the slope of $G_1\left( {\hat{\alpha }}\right)$ decreases. Let  correspond to the equilibrium in zone *B* under  and $\left( {\widehat{G}}_1(),{\widehat{G}}_2(),{\widehat{V}}_1,{\widehat{V}}_2\right)$—under $({\widehat{\rho }}_1,{\widehat{\rho }}_2)$, such that . Note that  for ${\hat{\alpha }}\in [0,r)$ and  for . ${\widehat{G}}_1$ and  intersect exactly once in (*r*, 1), since they have equal expectations, $\alpha _{1,0}$. Thus, the supremum of continuous support corresponding to ${\widehat{G}}_1$ must be higher, . Therefore $\exists !\alpha ^{\dagger }\in (r,1)$ such that for ${\hat{\alpha }}<\alpha ^{\dagger }$, ; and for ${\hat{\alpha }}>\alpha ^{\dagger }$, . Thus, , and strictly so for ${\hat{\alpha }}\in (r,1)$. Therefore, , and the increase of $\rho _1$ in *B* increases 1’s distribution in the *SOSD*-sense.

The slope of $G_2$ in the increasing part is $1/V_1$. Since ,  and ${\widehat{G}}_2$ is steeper than  when both are continuously increasing. ${\widehat{G}}_2$ is increasing on a broader range than , since . For ${\widehat{G}}_2$ and  to intersect exactly once in (*r*, 1), it must hold that  if $x<r$. Hence, , where $\frac{r+u_1}{V_1}=\frac{r+(\rho _2-\rho _1+\rho _1^2V_1-\rho _2^2V_2)/(\rho _1^2)}{V_1}$ is the value of $G_2(x)$ in $x\in [0,r)$. Again, we conclude that there exists a unique $\alpha ^{\ddagger }\in (r,1)$: ; ; and , with strict inequality for all ${\hat{\alpha }}\in (r,1)$, i.e. .

Consider now the preferences of the investor. For a fixed realization of posteriors, $\left( {\hat{\alpha }}_1,{\hat{\alpha }}_2\right) =(x,y)$, the payoff of the investor is $v^P(x,y)=\left[ \max \{x,y\}-r\right] _+$, where $[.]_+$ denotes the positive part. The ex-ante expected payoff is, then,

$$\begin{aligned} {\mathbb {E}}v^P={\mathbb {E}}\left[ \max \left\{ {\hat{\alpha }}_1,{\hat{\alpha }}_2\right\} -r\right] _+=\int _{0}^{1}\left( \int _{0}^1\left[ \max \{x,y\}-r\right] _+dG_1(x)\right) dG_2(y). \end{aligned}$$

Note that $v^P(x,y)$ is a convex function of *x* for any *y*, while ${\mathbb {E}}_{{\hat{\alpha }}_1}v^P({\hat{\alpha }}_1,y)$ is a convex function of *y*. Thus, using the fact that risk-loving agents prefer second-order dominated lotteries, we have

We conclude that the investor prefers the equilibrium under  to that under ${\widehat{\rho }}$. Moreover, it can be shown that the preference is strict because the expression $\int _{0}^1\left[ \max \{x,y\}-r\right] _+dG_1(x)$ is strictly convex for a non-zero measure of *y* and  for all $y\in (0,1)$. $\square$

### Proof of Lemma 2

#### Proof

Consider the system (A.3)–(A.4) which defines relation between expenditures/priors and values in zone $B^{\prime }$:

Transform it and include the equation that defines $B^{\prime }$, $\rho _1^2V_1-\rho _1-\rho _2^2V_2+\rho _2=0$, to have

B.1 $$\begin{aligned} 2\rho _1^2&\alpha _{1,0}V_2-2\rho _1(V_2-1)-\rho _1^2r^2(2t-1)-\left( \rho _2V_2-1\right) ^2=0, \end{aligned}$$

B.2 $$\begin{aligned} 2\rho _1^2&\alpha _{2,0}V_1-2\rho _1(V_2\rho _2-1)-\rho _1^2(2V_1-r^2)-(\rho _2V_2-1)^2=0, \end{aligned}$$

B.3 $$\begin{aligned} \rho _1^2&V_1-\rho _1-\rho _2^2V_2+\rho _2=0, \end{aligned}$$

a system, which we will denoted as $\Xi ^{\prime }$. As in the previous proof, use the implicit function approach to determine the signs of $\frac{dV_1}{d\rho _1}$ and $\frac{dV_2}{d\rho _1}$ that follow from the system above. Treat the variables $(V_1,V_2,t)$ as endogenous, and $\rho _1$ and $\rho _2=1-\rho _1$ as exogenous. The matrix of derivatives of the system above with respect to $(V_1,V_2,t)^T$ is

$$\begin{aligned} J^{\prime }=\begin{pmatrix} 0 &{} 2\left( \rho _2-\rho _1(1-\alpha _{1,0}\rho _1) -\rho _2^2V_2\right) &{} -2\rho _1^2r^2\\ -2(1-\alpha _{2,0})\rho _1^2 &{} -2\rho _2\left( \rho _1+\rho _2V_2-1\right) &{} 0\\ \rho _1^2 &{} -\rho _2^2&{} 0 \end{pmatrix}. \end{aligned}$$

The derivative of system $\Xi ^{\prime }$ with respect to $\rho _1$ (again, keeping in mind that $d\rho _2-d\rho _1$) is

B.4 $$\begin{aligned} \frac{\partial \Xi ^{\prime }}{\partial \rho _1}= \begin{pmatrix} 2\left( 1+\rho _1r^2(1-2t)+V_2(\rho _2V_2-2(1-\rho _1\alpha _{1,0}))\right) \\ 2\left( 1-\rho _2V_2(2-V_2)+r^2\rho _1-2\rho _1V_1(1-\alpha _{2,0})\right) \\ 2\left( \rho _1V_1+\rho _2V_2-1\right) \end{pmatrix}. \end{aligned}$$

We are interested in the first two elements of $-J^{\prime -1}\times \frac{\partial \Xi ^{\prime }}{\partial \rho _1}$, which will give us $dV_1/d\rho _1$ and $dV_2/d\rho _1$. It can be checked that

$$\begin{aligned} \frac{dV_1}{d\rho _1}&=\frac{-2\rho _2(V_2-1)^2V_1}{\rho _1\left( (\rho _2-\rho _1)(V_2-1)^2+\rho _1^2(r^2+V_2^2)\right) },\\ \frac{dV_2}{d\rho _1}&=\frac{2(V_2-1)(r^2+V_2)\rho _1}{\rho _2\left( (\rho _2-\rho _1)(V_2-1)^2+\rho _1^2(r^2+V_2^2)\right) }, \end{aligned}$$

where we also used the fact that $V_1=\left( \rho _2^2V_2+\rho _1-\rho _2\right) /\rho _1^2$ and the value of $\alpha _{2,0}$ from (A.4) for some further simplification.

The sign of $\frac{dV_1}{d\rho _1}$ is negative: the numerator is negative, given $V_2\geqslant 1$; the denominator is positive at $V_2=1$ and is increasing in $V_2$ if $V_2>1$. Similarly, $\frac{dV_2}{d\rho _1}$ is positive.

We can now conduct the comparative statics of $G_2$. Letting  and relying on the single-crossing property of  and ${\widehat{G}}_2(x)$, as we did in the proof B.1 of Lemma 1, we can conclude that , as .

As for $G_1$, the value of ${\widehat{G}}_1(x)$ at $x=r$ and the slope in the range $(r,(1-{\widehat{\rho }}_2{\widehat{V}}_2)/{\widehat{\rho }}_1)$ are lower than those for . From the single-crossing, it must hold that . Hence, .

Similarlly to the proof of the previous lemma, the principal prefers it, when both distributions are dominated in the *SOSD*—sense, hence, he prefers ${\widehat{\rho }}$ to . $\square$

### Proof of Lemma 3

#### Proof

Part (iii) of Lemma 3 follow directly from the definition of zone *D*.

For part (ii), the decrease of the upper bound on $\alpha _{2,0}$ also follows directly from the definition of zone $B^{\prime }$. In order to prove the the statement in terms of bounds on $\alpha _{1,0}$, consider again the system $\Xi$ from B.1–B.3. Apply the implicit function theorem to evaluate $\frac{d\alpha _{1,0}}{d\rho _1}$, treating $\alpha _{1,0}$, $V_1$, $V_2$ as endogenous variables, $\rho _1$ as an exogenous variable, and keeping $\alpha _{2,0}$ fixed, and allowing $t=0$ for the lower bound and $t=1$ for the upper bound. The matrix of derivatives of the system with respect to $(\alpha _{1,0},V_1,V_2)^T$ is

$$\begin{aligned} J^{\prime }=\begin{pmatrix} 2\rho _1^2V_2&{}0&{} 2\left( \rho _2-\rho _1(1-\alpha _{1,0}\rho _1) -\rho _2^2V_2\right) \\ 0 &{} -2(1-\alpha _{2,0})\rho _1^2 &{} -2\rho _2(\rho _1+\rho _2V_2-1)\\ 0 &{} \rho _1^2&{} -\rho _2^2\\ \end{pmatrix}, \end{aligned}$$

and the derivative of the system with respect to $\rho _1$ the same as before, in (B.4). We need the first element of $-J^{\prime ,-1}\times \frac{\partial \Xi ^{\prime }}{\partial \rho _1}$, which is

B.5 $$\begin{aligned} \frac{d\alpha _{1,0}}{d\rho _1}&=- \frac{(V_2-1)}{V_2^2\rho _2\rho _1^3((V_2-1)^2(\rho _2-\rho _1)+\rho _1^2(V_2^2+r^2))}\times \chi ,\nonumber \\ \chi&\doteq \left[ V_2(V_2-1)^3(\rho _2-3\rho _1)\right. \nonumber \\&\quad +\left. (V_2-1)\rho _1^2\left( r^2(\rho _1-\rho _2)+V_2\left( 4-V_2\left( 5+6\rho _2-2V_2(1+2\rho _2)\right) \right) \right) \right. \nonumber \\&\left. \rho _1^4\left( r^4(2t-1)+2r^2(t-1)V_2-(2-V_2)V_2^3\right) \right] , \end{aligned}$$

where we plugged in the values of $\alpha _{1,0}$ and $\alpha _{2,0}$ from A.3 and A.4 and $V_1$ from B.3. Let us determine the sign of $\frac{d\alpha _{1,0}}{d\rho _1}$ given $V_2\in \left[ 1,\frac{1-\rho _1r}{1-\rho _1}\right]$. The denominator is positive similarly to B.2.

To determine the sign of $\chi$, first proceed with

taking the second derivative of it with respect to $V_2$. We have,

$$\begin{aligned} \frac{\partial ^2\chi }{\partial V_2^2}&=6(V_2-1)\rho _2^2\left( 2\rho _2^2V_2+\rho _1-\rho _2\right) \geqslant 6(V_2-1)\rho _2^2(2\rho _2^2+\rho _1-\rho _2)\\&=6(V_2-1)\rho _2^2(\rho _2^2+\rho _1^2)\geqslant 0. \end{aligned}$$

This implies, that $\frac{\partial \chi }{\partial V_2}$ is increasing for $V_2\in \left[ 1,\frac{1-\rho _1r}{1-\rho _1}\right]$, and achieves its maximum in that range under $V_2=\frac{1-\rho _1r}{1-\rho _1}$. Check that $\frac{\partial \chi }{\partial V_2}$ attains a non-positive value given $V_2=1$:

$$\begin{aligned} \frac{\partial \chi }{\partial V_2}|_{V_2=1}&= \rho _1^2\left( -1-2\rho _2\rho _1+r^2(-1+2\rho _1(1-(1-t)\rho _1))\right) \\&\leqslant \rho _1^2\left( -1-2\rho _2\rho _1+1\times (-1+2\rho _1(1-(1-1)\rho _1))\right) \\&=-2\rho _1^2\rho _2^2\leqslant 0. \end{aligned}$$

This implies that either $\chi$ attains a local minimum in $V_2$ for $V_2\in \left[ 1,\frac{1-\rho _1r}{1-\rho _1}\right]$ (this happens if $\frac{\partial \chi }{\partial V_2}$ is positive for $V_2=\frac{1-\rho _1r}{1-\rho _1}$); or $\chi$ is decreasing in $V_2$ in that range. In either case, it would be sufficient to check that $\chi \leqslant 0$ for $V_2\in \left\{ 1,\frac{1-\rho _1r}{1-\rho _1}\right\}$, to conclude that $\chi \leqslant 0$ in the whole interval $V_2\in \left[ 1,\frac{1-\rho _1r}{1-\rho _1}\right]$. Doing so yields indeed $\chi |_{V_2=1}\leqslant 0$, and $\chi |_{V_2=\frac{1-\rho _1r}{1-\rho _1}}\leqslant 0$, as desired.

Overall, $\chi \leqslant 0$ and, thus, $\frac{d \alpha _{1,0}}{d\rho _1}\geqslant 0$ for $t\in \{0,1\}$. Hence, we have proven the part (*ii*) of Lemma 3. The part (*i*) follows, because the upper bound of *B* on $\alpha _{1,0}$ coincides with the lower bound of *D* on $\alpha _{1,0}$; and upper bound of *B* on $\alpha _{2,0}$ coincides with the upper bound of $B^{\prime }$ on $\alpha _{1,0}$. $\square$

## Equilibrium in *B* under $\rho _1=0$

The mapping established in this paper is not well-behaved for $\rho _1=0$. However, check that the following strategy profile constitutes an equilibrium under $\rho _1=0$ and $\alpha _{1,0}\in [\frac{1+r^2}{2},1-r+r^2)$, $\alpha _{2,0}\geqslant r$:

$$\begin{aligned} G_1(x)= {\left\{ \begin{array}{ll} 0, &{}\quad \text { if }x<r\\ \max \{x,{\overline{x}}(\alpha _{2,0})\}, &{}\quad \text { if }r\leqslant x<1 \\ 1,&{}\quad \text { if }1\leqslant {\hat{\alpha }}_1; \end{array}\right. } \quad {\hat{\alpha }}_{2}={\left\{ \begin{array}{ll} 1,&{}\quad \text { w.p. } \alpha _{2,0},\\ 0,&{}\quad \text { w.p. } 1-\alpha _{2,0}, \end{array}\right. } \end{aligned}$$

where ${\overline{x}}(\alpha _{2,0})\doteq 1-\sqrt{r^2+\alpha _{2,0}-1}$. While other equilibria are possible under $\rho _1=0$, we choose to work with the one above because it coincides with the limiting behavior of entrepreneurs as $\rho _1\downarrow 0$. Note also that the investor’s payoff from this equilibrium is $\alpha _{1,0}+\alpha _{2,0}-\alpha _{1,0}\alpha _{2,0}-r$, same as in zones *D* and $D^{M}$.

## Investor-preferred tie-breaking rules

Consider all point outside of *A*, $A^{\prime }$, and the span of *C*. The points $(\alpha _{1,0},\alpha _{2,0})$ in $\left[ \frac{1+r^2}{2},1\right] \times \left( 0,\frac{1-r^2}{2}\right)$ belong to zone *B* and/or *D* under various values of $\rho _1$. For every point in *B*, the investor’s payoff is monotonically decreasing in $\rho _1$, and in *D*, the paoyff is at the local maximum level. Hence, “rule 0” is optimal in $(\alpha _{1,0},\alpha _{2,0})\in \left[ \frac{1+r^2}{2},1\right] \times \left( 0,\frac{1-r^2}{2}\right]$.

The points in $\left[ \frac{1-r^2}{2},\frac{1+r^2}{2}\right] ^2$ can only belong to $B^{\prime }$ and $B^{\prime M}$ under various values of $\rho _1$. There, it is optimal for a point to be on line ${\underline{\alpha }}_1(\alpha _{2,0},\rho _1,\rho _2)$. Hence, rule $\rho _1^{\star }$ is optimal.

Any point in $\left( \frac{1+r^2}{2},1\right) \times \left( \frac{1-r^2}{2},\frac{1+r^2}{2}\right)$ can belong to $B^{\prime }$ and $B^{\prime M}$ under some tie-breaking rule. So $\rho _1^{\star }$ is one of the local maximizers. Any point in $\left( \frac{1+r^2}{2},1\right) \times \left( \frac{1-r^2}{2},\frac{1+r^2}{2}\right)$ can also belong either to *B*, or *D*, or to both. Hence, “rule 0” is another local maximizer.

The region $\left( \frac{1+r^2}{2},1\right) ^2$ is similar to $\left( \frac{1+r^2}{2},1\right) \times \left( \frac{1-r^2}{2},\frac{1+r^2}{2}\right)$, but additionally, it can be spanned by zones $B^{M}$ and/or $D^M$. In region $B^M$, the investor’s payoff is increasing in $\rho _1$ and in $D^M$ it is at the local maximum level. Hence, “rule 1” is a third local maximizer in that region.

## Footnotes

1 I also provide this characterization in my thesis, Muratov (2019).

2 On that figure, $u_1\doteq \frac{\rho _2-\rho _1+\rho _1^2V_1-\rho _2^2V_2}{\rho _1^2}$.

3 Here, $t\in [0,1]$ is again a free parameter.

4 This system defines the expenditures in region *B*.

5 The analysis also applies to the case of $\alpha _{1,0}<\alpha _{2,0}$ if we switch the roles of $\rho _1$ and $\rho _2$.
